# Estimation of Potential Deaths Averted From Hypothetical US Income Support Policies

**DOI:** 10.1001/jamahealthforum.2022.1537

**Published:** 2022-06-10

**Authors:** Anton L. V. Avanceña, Nicholas Miller, Ellen Kim DeLuca, Bradley Iott, Amanda Mauri, Daniel Eisenberg, David W. Hutton

**Affiliations:** 1 Department of Health Management and Policy, School of Public Health, University of Michigan, Ann Arbor; 2 Department of Epidemiology, School of Public Health, University of Michigan, Ann Arbor; 3 School of Information, University of Michigan, Ann Arbor; 4 Now with School of Medicine, University of California, San Francisco; 5 Department of Political Science, University of Michigan, Ann Arbor; 6 Department of Health Policy and Management, Fielding School of Public Health, University of California, Los Angeles; 7 Department of Industrial and Operations Engineering, College of Engineering, University of Michigan, Ann Arbor

## Abstract

**Importance:**

Income has a negative, nonlinear association with all-cause mortality. Income support policies may prevent deaths among low-income populations by raising their incomes.

**Objective:**

To estimate the deaths that could be averted among working-age adults age 18 to 64 years with hypothetical income support policies in the US.

**Design, Setting, and Population:**

An open, multicohort life-table model was developed that simulated working-age adults age 18 to 64 years in the US over 5 to 40 years. Publicly available household income data and previous estimates of the income-mortality association were used to generate mortality rates by income group. Deterministic sensitivity analyses were conducted to evaluate the effect of parameter uncertainty and various model assumptions on the findings.

**Interventions:**

In addition to a no-intervention scenario, 4 hypothetical income support policies were modeled: universal basic income, modified LIFT Act, poverty alleviation, and negative income tax.

**Main Outcome and Measures:**

The main outcome was the number of deaths averted, which was calculated by subtracting the number of deaths experienced in the no-intervention scenario from the number of deaths experienced with the various income support policies.

**Results:**

Base-case assumptions used average mortality rates by age, sex, and income group, a 20-year time horizon, and a 3-year lag time. Universal basic income worth $12 000 per year per individual was estimated to avert the most deaths among working-age adults (42 000-104 000 per year), followed by a negative income tax that guaranteed an income of 133% of the federal poverty level (19 000-67 000 per year). A modified LIFT Act that provided $6000 to individuals with annual household incomes less than $100 000 was estimated to avert 17 000 to 52 000 deaths per year. A targeted approach that alleviated poverty was estimated to prevent 12 000 to 32 000 deaths among the lowest-income, working-age adult population. Results were most sensitive to several inputs and assumptions, primarily the income-based mortality rates, analytic time horizon, and assumed time lag between when a policy was implemented and when individuals experienced the mortality benefit of having higher incomes.

**Conclusions and Relevance:**

In this modeling study, 4 hypothetical income support policies were estimated to avert thousands of deaths among working-age US adults every year. Additional research is needed to understand the true association of income gains with mortality. Discussions about the costs and benefits of income support policies should include potential gains in health.

## Key Points

**Question:**

How many deaths among working-age US adults can hypothetical income support policies, such as universal basic income, the modified LIFT Act, poverty alleviation, and negative income tax, potentially avert?

**Findings:**

In this multicohort modeling study that simulated US adults age 18 to 64 years over 5 to 40 years, broad income support policies, like universal basic income, were estimated to avert the most deaths among working-age adults, although targeted approaches, like poverty alleviation, may also avert thousands of deaths among low-income populations. Results were sensitive to several inputs, primarily the income group–specific mortality rates used.

**Meaning:**

The results of this study suggest that income support policies may prevent thousands of deaths among working-age US adults.

## Teaser

This modeling study estimates the deaths that could be averted among working-age adults age 18 to 64 years with hypothetical income support policies in the US.

## Introduction

Referred to as a *cause of causes*, income is an established social determinant of health and is associated with various health outcomes, including mortality.^1^ Prior research has demonstrated that income and mortality have a negative, nonlinear association. Studies in the US have shown that adults with lower incomes experience all-cause mortality rates up to 2 times higher than their counterparts with higher incomes.^2,3,4^ Another study found that males and females at the top 1% and bottom 1% of the income distribution, respectively, have a 14-year and 10-year difference in life expectancy.^5^

Income support and other redistributive policies have the potential to eliminate poverty and improve population health.^6^ For example, the Earned Income Tax Credit has been associated with improved survival, self-reported health, and child development.^7^ Among older adults, Social Security income has been associated with declines in all-cause mortality.^8^ Other proposals, like raising the minimum wage and cash transfers, have also been associated with improved health and have gained public support.^9,10^ However, discussions about the effects of these policies have emphasized their economic and financial costs while leaving out their potential health equity benefits, partially because of the mixed evidence available to date.^11,12^

In this exploratory modeling study, we estimated the potential health benefits (in terms of deaths averted) of 4 hypothetical income support policies (universal basic income, modified LIFT Act, poverty alleviation, and a negative income tax) compared with a no-intervention scenario. Through several sensitivity analyses, we also sought to identify the most important considerations that might change the magnitude of the potential health benefits of income support policies.

## Methods

### Model Design

We developed a multicohort life-table model in Microsoft Excel that simulated an open or dynamic population of working-age adults age 18 to 64 years in the US (eMethods and eTable 1 in the Supplement). Life-table models are widely used in demography and decision analysis to estimate the longevity benefits of various interventions.^13^ The inputs and assumptions are summarized in Table 1.

**Table 1.** Summary of Modeling Approach and Assumptions

Characteristic	Description
Type of simulation model	Open, multicohort life-table model with 2 states (ie, dead and alive)
Population No. by income	2020 ASEC of the US Census Bureau
Population-specific mortality	Calculated using (1) all-cause mortality rates from the CDC, (2) income-mortality rate ratios reported in 2 studies using data from the NLMS and PSID, and (3) population weights from ASEC
Mortality benefit of income gains	Assumed to be equal to difference in mortality between original income group and new income group; evidence to date is mixed and contested in the literature
Lag time	Minimum of 3 y; changed to 5, 10, and 15 y in sensitivity analysis
Other key assumptions	(1) Income gains associated with mortality reductions; (2) magnitude of all-cause mortality rates is static over time; (3) changes in household income are captured by income bands; (4) new entrants to the model have the same income distribution as the original cohort of the same age

Abbreviations: ASEC, Annual Social and Economic Supplement; CDC, US Centers for Disease Control and Prevention; NLMS, National Longitudinal Mortality Study; PSID, Panel Study for Income Dynamics.

We followed best practices for modeling and decision analyses when possible, including Consolidated Health Economic Evaluation Reporting Standards (CHEERS) guidelines.^14,15^ For example, we used national estimates of the US population by household income, described the sources for and assumptions around intervention effects, and systematically evaluated the effect of parameter uncertainty on our findings. This modeling study qualified as unregulated research activity.^16^

### Policy Descriptions

We modeled 4 hypothetical policies that raised the total household income of eligible individuals (Table 2; eTable 15 in the Supplement). The first policy was universal basic income in which every adult received a monthly transfer of $1000, raising household income by at least $12 000 per year. The second policy was a smaller transfer of $500 per month for every adult with household incomes less than $100 000 per year, which is a modified version of the LIFT Act that Vice President Kamala Harris proposed while in Congress.^17^ In the third policy, all adults were lifted out of poverty by an income guarantee of at least 100% of the federal poverty level (FPL) for 1 individual (ie, $12 760 in 2019). Finally, the fourth policy was a hypothetical negative income tax, which guaranteed an income equal to 133% of the FPL and rewarded earned income up to a certain amount (eTable 16 in the Supplement).^18^

**Table 2.** Description of Modeled Policies

Policy	Description	Projected change in income, $	Population affected
Universal basic income	Unconditional basic income guarantee through flat-rate transfers regardless of current income	1000 per month (12 000 per year)	All working-age adults
Modified LIFT Act	$500 Monthly tax credit	500 per month (6000 per year)	Working-age adults earning less than $100 000 per year
Poverty alleviation	All adults are lifted out of poverty, defined as having incomes less than 100% of the FPL	Difference between current income and 100% of FPL	Working-age adults with incomes less than 100% of the FPL
Negative income tax	Conditional basic income guarantee in which adults are guaranteed an income at least 133% of the FPL, and each additional earned income is matched by the government at a rate of 50%	Variable depending on current income; up to 133% of FPL in transfers per year	Working-age adults with incomes up to 266% of FPL

Abbreviations: FPL, federal poverty level; LIFT, Livable Incomes for Families Today.

### Input Parameters

#### Population Numbers

We populated the life-table model with national population estimates by household income, sex, and age group from the 2020 Annual Social and Economic Supplement (ASEC; eTable 2 in the Supplement). Household income in the ASEC is defined as the total sum of all types of income, such as wages and salaries, self-employment income, and government benefits (all subsequent references to income in this study refer to household income). A detailed description of the ASEC is found in the eMethods in the Supplement and other sources.^19^

Population estimates from the ASEC are reported in multiyear age groups (eg, 18-21 and 22-24 years), which we handled by making 2 assumptions about the distribution of individuals across different ages. In the equal age assumption, we evenly divided individuals among the years within each age group. In the median age assumption, we assumed that individuals in each age group had the median age of the age group (median age assumption; eTable 3 in the Supplement).

#### Age-, Sex-, and Income Group–Specific Mortality Rates

We calculated all-cause mortality rates by age, sex, and income group for the model using data from the ASEC (eTables 5 and 6 in the Supplement),^19^ life tables from the US Centers for Disease Control and Prevention (CDC),^20^ and 2 observational studies on the nonlinear association between income and mortality.^2,3^ The 2 observational studies were identified through a systemic review process (eMethods and eTable 4 in the Supplement). Two sets of mortality rates were estimated from these 2 studies to use for the model.

The ASEC data were used to calculate population weights by income group, and the 2 observational studies were used to calculate income-specific mortality rates by using the estimated or modeled mortality rate ratios from each study. The first observational study used data from the National Longitudinal Mortality Study (NLMS)^2^ to estimate mortality rate ratios between income groups, and the second study used data from the Panel Study of Income Dynamics (PSID).^3^ To our knowledge, few randomized clinical trials and quasi experimental studies have explored the causal relationship between income gains or income support policies with mortality in the US, and most have been limited to specific subgroups (eg, low-income working adults, individuals of racial and ethnic minority groups) or short-term interventions,^7,11,12^ rendering them unsuitable for the current analysis, which is focused on long-term income support policies across many income groups. We acknowledge that the association between income gains or income support policies and reductions in mortality that this study depends on is uncertain and contested in the literature.^21,22^ We also recognize that these studies do not control for all confounding variables that may have important interactions with income and health. Nevertheless, we derived mortality rates for the model from 2 studies that used the best available US data to estimate the income association with health.

**Figure 1.**
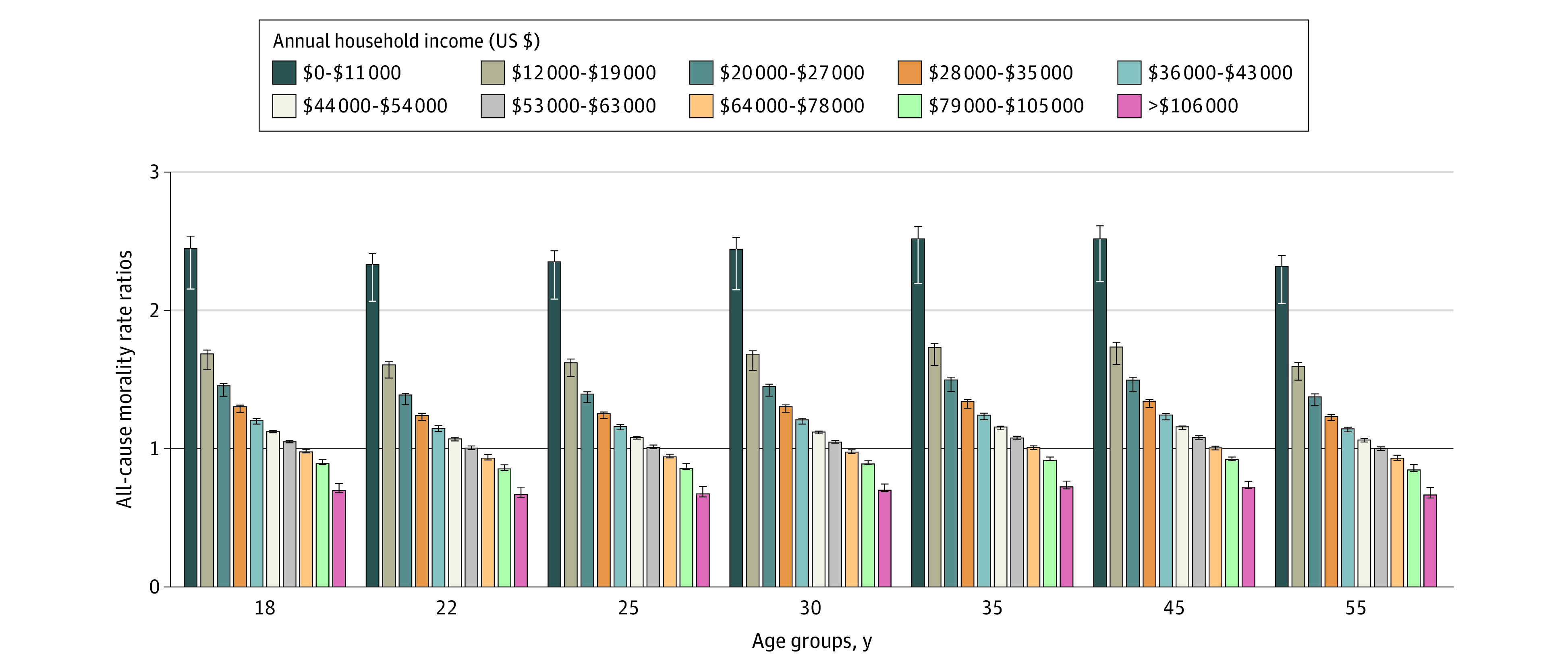
Estimated All-Cause Mortality Rate Ratios Among Females by Annual Household Income Group The rate ratios were calculated by dividing the mortality rates estimated using results from the National Longitudinal Mortality Study (NLMS) and Panel Study for Income Dynamics (PSID) by mortality rates reported in US Centers for Disease Control and Prevention (CDC) life tables for the US. The horizontal black line marks a rate ratio equal to 1. Error bars denote the estimated range of mortality rates within different scenarios. Household incomes are in thousands USD per year.

To facilitate comparisons between the NLMS and PSID, mortality rates were calculated using the same household income groups for both studies. Additionally, we assumed that the CDC life tables represented the weighted average of mortality rates for all income groups for each age (eMethods in the Supplement). We based this assumption on prior research that showed an income-mortality gradient in which lower-income individuals lived shorter lives compared with their higher-income counterparts.^4,5,11^ Figure 1 shows the estimated income-specific mortality rates for females across various ages, and eTables 7 to 10 in the Supplement present the income-specific mortality rates for all ages and sexes.

#### Effect of Modeled Policies

For each policy scenario (Table 2), we estimated the corresponding income gain for eligible individuals and updated the total household income (eMethods and eTable 18 in the Supplement). We then recalculated the number of individuals in each household income group and used those population numbers in the model. In this study, we assumed that individuals who moved from one household income group to another experienced the mortality rate associated with their new household income group. This is a major limitation, and we tested the effect of this assumption by conducting several sensitivity analyses that reduced or moderated the association of income gains with future mortality.

We treated additional income gained from different policies the same, although in practice various mechanisms through which supplemental income is distributed may have differential associations with health.^23^ We also assumed that any secular changes in household income were captured by the income ranges to which individuals were assigned. Finally, we assumed that new cohorts of individuals aged 18 years who entered the model after the first year had the same income distribution as those in the original population that was simulated. These simplifying assumptions may not reflect true income dynamics in the US, especially when income support policies are implemented.

### Analysis Plan

The main outcome of interest was the number of deaths averted among working-age adults age 18 to 64 years, which we calculated by taking the difference between the total deaths in the no-intervention scenario and the total deaths from each modeled policy. We focused our analysis on working-age adults to match the participants in the NLMS and PSID, although we recognize that income support policies, as well as Social Security and Medicare, may benefit individuals older than 65 years.^24,25^ In the base-case analysis, we applied the equal age assumption, used a 20-year time horizon, and assumed a 3-year lag between income gains and mortality benefit. Results were not discounted.

### Sensitivity Analyses

We conducted several deterministic sensitivity analyses. First, we varied the distribution of the population by age from the equal age assumption to the median age assumption. Second, we varied the time lag or delay between individuals experiencing income gains and experiencing the mortality rates of their new income group. Studies on the health effects of income support policies have documented health benefits after 3 years,^7,11,26^ so we used this in the base-case analysis. We varied this time lag in multiple scenario analyses using 5-year, 10-year, and 15-year lags. Third, we used 2 additional sets of mortality rates by age, sex, and income from the NLMS and PSID, which we labeled as high- and low-effect scenarios (eMethods and eTables 11-14 in the Supplement). These high-effect and low-effect scenarios reflected the uncertainty in the estimated association between income and mortality in each study and were derived from the 95% CIs. In the high-effect scenario, income had a larger effect on mortality, whereas in the low-effect scenario, income had a smaller effect on mortality (eTables 11-14 in the Supplement). Fourth, we changed the time horizon to 5, 10, 30, and 40 years, which represented different time horizons that policy makers may use when evaluating income support policies. Different combinations of the inputs and assumptions generated 180 estimates of the number of deaths averted for each modeled policy.

## Results

### Base-Case Results

**Figure 2.**
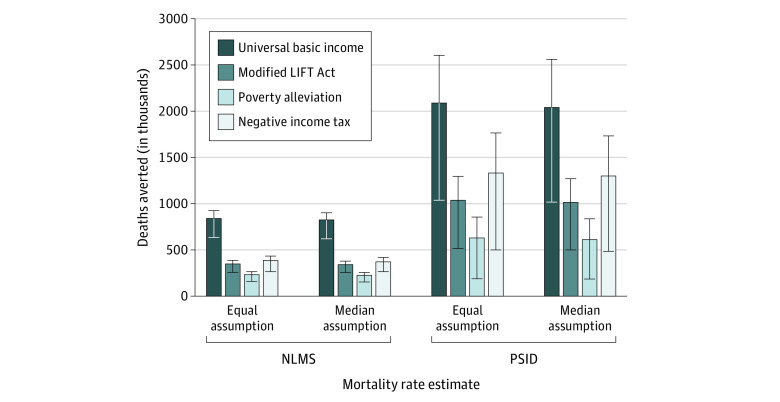
Base-Case Deaths Averted From Income Support Policies Among Working-Age US Adults Number of deaths averted (in thousands) from 4 hypothetical income support policies in the US within base-case assumptions and varied age assumption. The error bars denote the estimated range of deaths averted using different mortality rates from the National Longitudinal Mortality Study (NLMS) and Panel Study for Income Dynamics (PSID) studies.

The base-case results are shown in Figure 2. Universal basic income was associated with the most deaths averted among working-age adults using the NLMS (44 000 annual average deaths averted) and PSID mortality rates (104 000 annual average deaths averted). This was the expected result, because universal basic income shifted the most people from their current income group to the next highest income group (eFigure 2 in the Supplement), which was associated with a lower mortality rate. Negative income tax was associated with the next highest total number of deaths averted among working-age adults (19 000-67 000 annual average deaths averted using NLMS and PSID rates, respectively), followed by a modified LIFT Act (18 000-52 000 annual average deaths averted using NLMS and PSID rates, respectively). Poverty alleviation, which was the most limited and targeted of the interventions, was associated with the fewest number of deaths averted (12 000-32 000 annual average deaths averted using NLMS and PSID rates, respectively).

Larger gains in averted deaths were found when PSID mortality rates were used in the model across all policies (Figure 2), and this reflected the substantially higher mortality rates for the lowest-income groups that we estimated using that study compared with the NLMS (Figure 1). For example, the all-cause mortality rate among males aged 18 years in the lowest income group was estimated to be more than 2.4 times higher in the PSID than in the NLMS. Thus, if income policies move populations from low-income groups to higher-income groups, the benefits are more pronounced.

### Sensitivity Analysis

Detailed results of the sensitivity analyses are found in eTables 19 to 22 in the Supplement for all policies. Changing the age assumption did not significantly affect the results of comparable scenarios, particularly analyses that used long time horizons (Figure 2). This suggests that precision around the age of beneficiaries mattered less than other inputs in the model.

**Figure 3.**
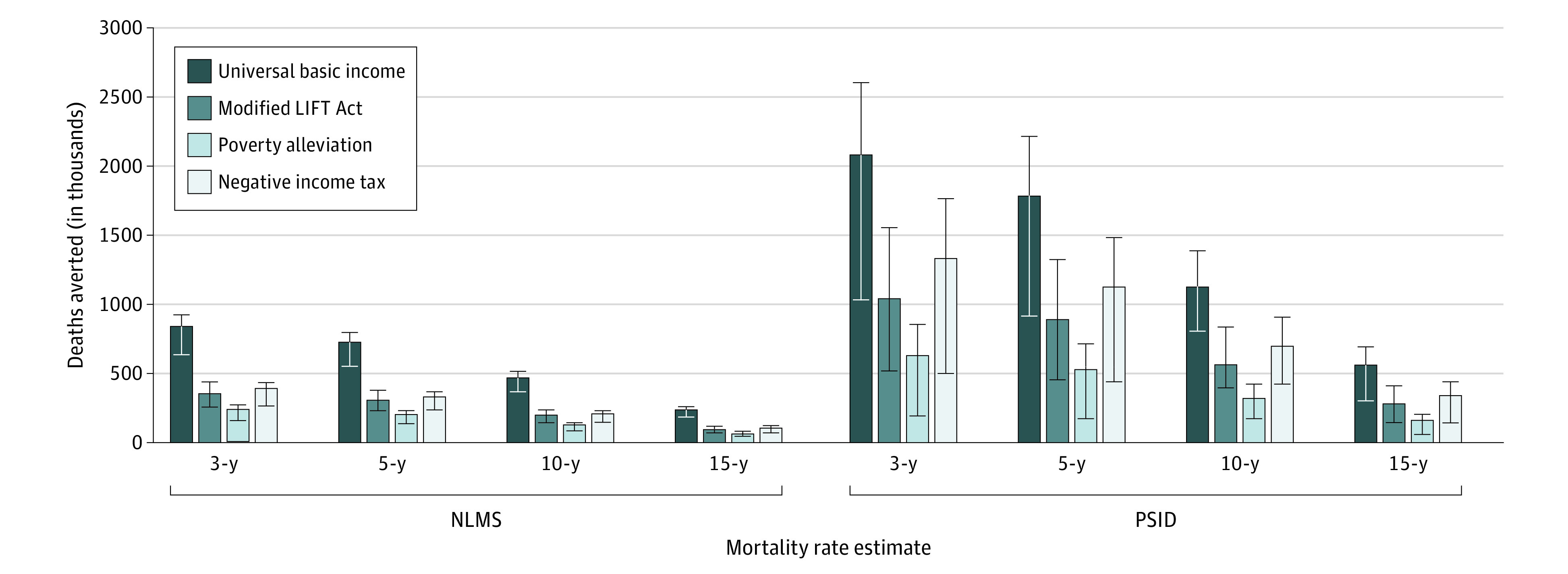
Deaths Averted From Income Support Policies Within Different Lag Times Number of deaths averted (in thousands) among working-age US adults from 4 hypothetical income support policies in the US using different lag times over a 20-year time horizon. The error bars denote the estimated range of deaths averted using different mortality rates (high effect vs low effect) from the National Longitudinal Mortality Study (NLMS) and Panel Study for Income Dynamics (PSID) studies while keeping other assumptions unchanged.

The most influential parameters in the model were the estimates of the income group-specific mortality rates (ie, NLMS vs PSID), assumed lag time, and analytic time horizon. The high-effect and low-effect scenarios, represented by error bars in Figure 2 and Figure 3, were also influential parameters; for example, use of the high-effect and low-effect mortality rate estimates was associated with a 8% to 24% difference in the deaths averted using NLMS and a 19% to 47% difference using the PSID for the same policy. Increasing the lag time between income gains and when mortality benefits are realized was associated with a reduced number of deaths averted across all policies (Figure 3).

## Discussion

This modeling study of 4 hypothetical income support policies estimated tens of thousands of deaths that could be averted annually for working-age US adults. Sensitivity analyses suggested that the results were most sensitive to the policy being modeled, income-based mortality rates, and assumed time lag between income gains and mortality benefits.

This study is one of several simulation model-based analyses that estimate the negative effects of low incomes, as well as the benefits of income support and other fiscal policies in the US. One study found that a progressive tax reform plan could yield 31 000 fewer deaths per year and substantially more if revenues were redistributed to lower-income individuals.^27^ One study estimated that 4% to 8% of premature deaths could have been prevented by increasing the minimum wage in New York, New York, from $9 to $15.^28^ Similar to our analysis, another study evaluated the mortality benefits of hypothetical income support policies, such as moving people out of poverty; depending on the policy, providing low-income households with more income was associated with a 3% to 8% reduction in mortality.^2^ The current study adds to this literature by exploring income support policies that to our knowledge have not been previously evaluated in the US context and estimating the benefits of these policies across the entire income distribution.

A smaller body of literature has evaluated the benefits and costs of income-based policies concurrently. For example, a cost-effectiveness analysis found that for the average Earned Income Tax Credit recipient, the program costs $7786 per quality-adjusted life year gained. Although a specific cost-effectiveness threshold has not been established in the US, this is substantially lower than the cost-effectiveness thresholds commonly used (ie, $100 000-$150 000 per quality-adjusted life year gained) to assess a health intervention’s efficiency.^29^ Future studies can compare the results of the present study with the costs of implementing income support policies to understand their relative efficiency. Recent estimates of the annual financial costs of universal basic income ranged from $2.49 to $3.03 trillion depending on the size of the transfer,^30,31^ while the LIFT Act has been estimated to cost $300 billion per year.^17^ By contrast, poverty alleviation and a negative income tax are substantially more targeted interventions and will cost less.^18^ Using these annual financial costs and this study’s base-case results (Table 2), each death averted is roughly estimated to cost around $16 to $43 million with universal basic income and $3.7-12 million with the LIFT Act. In comparison, the value of a statistical life used by the US Environmental Protection Agency is $7.4 million. However, these rough cost estimates do not reflect any potential reductions in health care spending that are associated with improvements in population health like those modeled in this study, or potential productivity gains from longevity among working-age adults.^11^

### Limitations

There are several limitations to this study, which we discuss in greater detail in the eMethods in the Supplement. First, we used cross-sectional estimates of the nonlinear association between income gains and mortality, and we assumed that individuals who received additional income experienced reductions in their mortality risk after a lag. Although some argue that the detrimental effects of having a low income are hard to reverse because of how income-based health risks are embodied and embedded (eg, exposure to toxins), previous studies have found that income gains are associated with reductions in mortality in the US^7^ and elsewhere.^32^ Additionally, a life course perspective on income and health suggests that additional resources at critical periods in life can be associated with improvements in health, including survival.^33^ For example, studies have found an association of Social Security benefits with improved health and reduced mortality among older adults.^8^ Future analyses should rely on causal estimates when they become available.

Second, given ASEC household income data, we had to make simplifying assumptions to reasonably estimate the total household income following each income support policy. Third, we did not model the effect of potential mechanisms, such as progressive taxation, that may be used to fund income support policies. We also did not model the association of these policies with jobs, prices, and other economic domains, and a general equilibrium analysis that considers the association of income support policies with the whole system may elucidate these issues. Fourth, we modeled household and not personal income, so any income increases that are associated with age were not included. Additionally, we assumed that any secular changes in household incomes were captured by the income bands, which is partially supported by evidence that intragenerational economic mobility in the US has remained stable since the 1980s.^34^ Fifth, because the NLMS and PSID focused on adult mortality, we excluded children in this study. Sixth, unlike previous studies,^27,35^ we did not model the association of income inequality with mortality, although there is evidence that the magnitude of income inequality is positively and independently associated with mortality and other poor health outcomes.^36^ Seventh, we assumed that all-cause mortality rates do not change over time; in reality, death rates have generally decreased in the US, although increases have also been noted, particularly in working-age adults even before the COVID-19 pandemic.^37^ Finally, we focused exclusively on income in this study and did not include the effects of other measures of socioeconomic position, such as education, employment, or wealth, which also have documented associations with health.^35^ Relatedly, the estimates of the income-mortality gradient we relied on may be subject to confounding from unmeasured factors associated with income and health. Future studies should examine the independent effects of these social determinants and their intersections, including race and ethnicity, which have been shown to be a significant predictor of mortality in the US.^4^

## Conclusions

In this modeling study, hypothetical income support policies were estimated to prevent thousands of deaths among working-age US adults. Despite decades of research that has demonstrated that income is an important determinant of health, discourse around income support policies has disproportionately emphasized their economic benefits and costs, with little to no focus on the health benefits that these interventions might provide.^6^ While substantial gaps in evidence exist, this exploratory analysis potentially broadens the view by including population health in public policy evaluations.

## Notes

Supplement.
eMethods.
**eTable 1. **Life-table model for a single cohort with starting age, household income level, and gender **eTable 2. **US population numbers (in thousands) from the 2020 Current Population Survey by household income group, age group, and gender **eTable 3. **Median ages used in the median assumption **eTable 4. **Inclusion and exclusion criteria **eTable 5. **Proportion of males in each National Longitudinal Mortality Study household income group by age and gender using data from the 2020 Current Population Survey **eTable 6. **Proportion of females in each National Longitudinal Mortality Study household income group by age and gender using data from the 2020 Current Population Survey **eTable 7. **Base estimate of age- and household income group-specific mortality rates among males estimated using data from National Longitudinal Mortality Study **eTable 8. **Base estimate of age- and household income group-specific mortality rates among females estimated using data from National Longitudinal Mortality Study **eTable 9. **Base estimate of age- and household income group-specific mortality rates among males estimated using data from Panel Study of Income Dynamics **eTable 10. **Base estimate of age- and household income group-specific mortality rates among females estimated using data from Panel Study of Income Dynamics **eTable 11.** Mortality rates estimated using data from the National Longitudinal Mortality Survey under high-effect scenario **eTable 12. **Mortality rates estimated using data from the National Longitudinal Mortality Survey under low-effect scenario **eTable 13. **Mortality rates estimated using data from the Panel Study of Income Dynamics under high-effect scenario **eTable 14. **Mortality rates estimated using data from the Panel Study of Income Dynamics under low-effect scenario **eTable 15. **Modeled policy scenarios **eTable 16. **Effect of hypothetical negative income tax policies **eTable 17. **Subgroup household incomes (in 2019 US$) **eFigure. **Distribution of the US population by household income under different scenarios **eTable 18. **Transitions between household income groups following implementation of modeled policies **eTable 19. **Deaths averted from Policy 1 (Universal basic income) under various assumptions (in thousands) **eTable 20. **Deaths averted from Policy 2 (Modified LIFT Act) under various assumptions (in thousands) **eTable 21. **Deaths averted from Policy 3 (Poverty alleviation) under various assumptions (in thousands) **eTable 22. **Deaths averted from Policy 4 (Negative income tax) under various assumptions (in thousands)
eReferences.
