# Supplementary material for: Estimation of Potential Deaths Averted From Hypothetical US Income Support Policies
Source: JAMA Health Forum. 2022 Jun 10;3(6):e221537. doi: 10.1001/jamahealthforum.2022.1537 (PMC9187947; doi:10.1001/jamahealthforum.2022.1537)
Supplement: Supplement. — eMethods. eTable 1. Life-table model for a single cohort with starting age, household income level, and gender eTable 2. US population numbers (in thousands) from the 2020 Current Population Survey by household income group, age group, and gender eTable 3. Median ages used in the median assumption eTable 4. Inclusion and exclusion criteria eTable 5. Proportion of males in each National Longitudinal Mortality Study household income group by age and gender using data from the 2020 Current Population Survey eTable 6. Proportion of females in each National Longitudinal Mortality Study household income group by age and gender using data from the 2020 Current Population Survey eTable 7. Base estimate of age- and household income group-specific mortality rates among males estimated using data from National Longitudinal Mortality Study eTable 8. Base estimate of age- and household income group-specific mortality rates among females estimated using data from National Longitudinal Mortality Study eTable 9. Base estimate of age- and household income group-specific mortality rates among males estimated using data from Panel Study of Income Dynamics eTable 10. Base estimate of age- and household income group-specific mortality rates among females estimated using data from Panel Study of Income Dynamics eTable 11. Mortality rates estimated using data from the National Longitudinal Mortality Survey under high-effect scenario eTable 12. Mortality rates estimated using data from the National Longitudinal Mortality Survey under low-effect scenario eTable 13. Mortality rates estimated using data from the Panel Study of Income Dynamics under high-effect scenario eTable 14. Mortality rates estimated using data from the Panel Study of Income Dynamics under low-effect scenario eTable 15. Modeled policy scenarios eTable 16. Effect of hypothetical negative income tax policies eTable 17. Subgroup household incomes (in 2019 US$) eFigure. Distribution of the US population by household income u [file jamahealthforum-e221537-s001.pdf]

Supplemental Online Content

Avanceña ALV, Miller N, Kim DeLuca E, et al. Estimation of potential deaths averted from hypothetical US income support policies. *JAMA Health Forum*. 2022;3(6):e221537.
doi:10.1001/jamahealthforum.2022.1537

eMethods.
eTable 1. Life-table model for a single cohort with starting age = x, household income level = i, and gender = g
eTable 2. US population numbers (in thousands) from the 2020 Current Population Survey by household income group, age group, and gender
eTable 3. Median ages used in the median assumption
eTable 4. Inclusion and exclusion criteria
eTable 5. Proportion of males in each National Longitudinal Mortality Study household income group by age and gender using data from the 2020 Current Population Survey
eTable 6. Proportion of females in each National Longitudinal Mortality Study household income group by age and gender using data from the 2020 Current Population Survey
eTable 7. Base estimate of age- and household income group-specific mortality rates among males estimated using data from National Longitudinal Mortality Study
eTable 8. Base estimate of age- and household income group-specific mortality rates among females estimated using data from National Longitudinal Mortality Study
eTable 9. Base estimate of age- and household income group-specific mortality rates among males estimated using data from Panel Study of Income Dynamics
eTable 10. Base estimate of age- and household income group-specific mortality rates among females estimated using data from Panel Study of Income Dynamics
eTable 11. Mortality rates estimated using data from the National Longitudinal Mortality Survey under high-effect scenario
eTable 12. Mortality rates estimated using data from the National Longitudinal Mortality Survey under low-effect scenario
eTable 13. Mortality rates estimated using data from the Panel Study of Income Dynamics under high-effect scenario
eTable 14. Mortality rates estimated using data from the Panel Study of Income Dynamics under low-effect scenario
eTable 15. Modeled policy scenarios
eTable 16. Effect of a hypothetical negative income tax policies
eTable 17. Subgroup household incomes (in 2019 US$)
eFigure. Distribution of the US population by household income under different scenarios
eTable 18. Transitions between household income groups following implementation of modeled policies
eTable 19. Deaths averted from Policy 1 (Universal basic income) under various assumptions (in thousands)
eTable 20. Deaths averted from Policy 2 (Modified LIFT Act) under various assumptions (in thousands)
eTable 21. Deaths averted from Policy 3 (Poverty alleviation) under various assumptions (in thousands)
eTable 22. Deaths averted from Policy 4 (Negative income tax) under various assumptions (in thousands)
eReferences.

This supplemental material has been provided by the authors to give readers additional information about their work.

© 2022 Avanceña ALV et al. *JAMA Health Forum.*

eMethods.

ACRONYMS AND ABBREVIATIONS

ASEC	Annual Social and Economic Supplement
CDC	Centers for Disease Control and Prevention
CPI	Consumer Price Index
CPS	Current Population Survey
EITC	Earned Income Tax Credit
FPL	federal poverty level
LIFT	Livable Incomes for Families Today
NIT	negative income tax
NLMS	National Longitudinal Mortality Study
PSID	Panel Study of Income Dynamics
UBI	universal basic income

VARIABLES AND INDICES

**Variable**	
l	population number
p	proportion of population in income group
q	mortality rate
r	mortality incident rate ratios by income group (from NLMS)
s	scenario or policy
x	age
y	year
z	all-cause mortality rate (from CDC life table)
φ	annual household income
Δ	change in mortality risk for each unit of income gain (from PSID)
**Index (possible values)**	
a	scenario or policy (0-4)
g	gender (1-2)
i	household income group (1-10)
j	cycle number (5-40)
k	knot (1)
x	age (18-64)

A. Model overview

We developed an open, multi-cohort life-table model to estimate survival and mortality among working-age adults 18-64 years in the US (see example in eTable 1). Life-table models are used in demography to estimate the life expectancy of a population given age-specific mortality rates[1], and increasingly life-table models (particularly multi-state life-table models) are used in decision analysis to estimate the costs and benefits of various health interventions.[2,3] We used population numbers (l_{igx}) from the US Census Bureau and we calculated age-, gender-, and income-specific mortality rates (q_{igx}) by applying previously published associations between income and mortality in the literature to all-cause mortality rates from US life tables published by the Centers for Disease Control and Prevention (CDC).

© 2022 Avanceña ALV et al. *JAMA Health Forum.*

eTable 1. Life-table model for a single cohort with starting age = x, household income level = i, and gender = g

Year	Age	Alive at year y	Mortality rate	Deaths
1	x	l_{igx}	q_{igx}	$l_{igx} * q_{igx}$
2	$x+1$	$l_{igx} - (l_{igx} * q_{igx})$	q_{igx+1}	$l_{igx+1} * q_{igx+1}$
…	…	…	…	…
y	$x+y$	$l_{igx} - \sum_{j=0}^{y-1}(l_{igx+j} * q_{igx+j})$	q_{igx+y}	$l_{igx+y} * q_{igx+y}$

The model runs for 5, 10, 20, 30, and 40 cycles or years, which represent different analytical time horizons that policymakers might be interested in when evaluating various income support policies. After the first year, new 18-year-olds are added to the simulated population (we refer to these as "new entrants"). We used the model to estimate the number of deaths averted from four hypothetical income support policies (s_{1-4}) compared to a no-intervention or status-quo scenario (s_0) where no income support policies are implemented. To calculate the deaths averted, we subtracted the estimated deaths under an income support policy scenario from the no-intervention scenario. The model was programmed in Microsoft Excel (Microsoft Corp, Redmond, Washington, USA).

B. Population numbers

We populated our life-table model with population estimates from the 2020 Annual Social and Economic Supplement (ASEC) of the Community Population Survey (CPS), which is administered by the US Census Bureau for the Bureau of Labor Statistics of the US Department of Labor. The CPS, which is a monthly survey, is the primary source of national employment statistics (e.g., employment rate), as well as of information on selected social and economic indicators in the US.[4] The ASEC is one of several CPS addendums and contains questions about demographics, labor force participation, work experience, income, noncash benefits, health insurance coverage, and migration, and it is the basis for national poverty rate.[5,6]

The CPS uses a probability-based sample of households in all 50 US states and the District of Columbia. Residents aged 15 and over are eligible to participate in the survey, provided they are not in the armed forces or institutionalized in correctional or long-term care facilities. The 2020 ASEC includes data from 60 400 household interviews (out of 79 400 eligible households) covering 157 880 unweighted individuals.[6] Data from the ASEC sample is used to estimate national-level statistics following procedures described elsewhere.[6]

We used population numbers by household income group, age group, and gender from the 2020 ASEC, which asked respondents about their total household income in 2019 (eTable 2).[7] Total household income (recoded as variable HHINC in ASEC data) is defined as "the arithmetic sum of all amounts received by all income recipients in the household," including money wages and salaries, net of income from self-employment, and income other than earnings.[6] We used aggregated data across all races and ethnicities.

© 2022 Avanceña ALV et al. *JAMA Health Forum.*

eTable 2. US population numbers (in thousands) from the 2020 Current Population Survey by household income group, age group, and gender

	**Males**							**Females**						
**Household income group (2019 US$)**	18-21 years	22-24 years	25-29 years	30-34 years	35-44 years	45-54 years	55-64 years	18-21 years	22-24 years	25-29 years	30-34 years	35-44 years	45-54 years	55-64 years
Under 5 000	149	148	211	151	273	297	422	152	186	275	187	333	358	576
5 000 to 9 999	123	71	80	69	184	159	288	98	95	215	147	199	265	470
10 000 to 14 999	158	94	141	128	285	266	480	157	124	181	202	320	360	756
15 000 to 19 999	134	141	163	130	321	306	566	203	166	232	205	417	483	579
20 000 to 24 999	201	150	283	273	325	361	620	185	194	372	282	450	456	730
25 000 to 29 999	250	190	324	278	466	410	543	271	239	364	325	622	490	635
30 000 to 34 999	289	229	437	352	535	440	580	274	281	408	396	597	554	793
35 000 to 39 999	257	183	328	319	510	440	526	260	242	380	343	631	516	624
40 000 to 44 999	269	231	431	487	624	533	676	198	249	440	447	722	533	861
45 000 to 49 999	299	254	398	368	639	518	722	276	254	323	367	663	645	808
50 000 to 54 999	257	208	471	431	753	555	610	268	295	507	439	684	553	700
55 000 to 59 999	257	248	441	374	619	535	591	256	256	393	358	628	598	629
60 000 to 64 999	270	295	503	400	644	626	573	262	243	469	356	668	678	730
65 000 to 69 999	288	213	431	377	560	452	573	263	216	392	375	591	598	579
70 000 to 74 999	262	177	399	371	728	537	586	218	157	459	324	656	492	629
75 000 to 79 999	190	178	395	436	607	521	580	198	171	349	434	589	558	606
80 000 to 84 999	220	187	409	443	531	623	505	184	186	435	367	603	524	601
85 000 to 89 999	231	198	403	341	527	524	493	220	177	308	302	512	535	584
90 000 to 94 999	193	162	349	294	538	457	527	213	157	349	332	555	537	583
95 000 to 99 999	173	157	349	370	599	481	500	219	137	324	337	553	466	554
100 000 to 104 999	194	122	355	332	643	511	434	177	168	342	281	594	506	516
105 000 to 109 999	143	181	256	309	529	432	422	196	114	292	294	494	416	478
110 000 to 114 999	160	128	285	246	564	430	469	124	96	277	253	527	451	492
115 000 to 119 999	164	113	262	315	448	429	382	161	102	232	246	432	393	386
120 000 to 124 999	235	151	279	271	516	521	437	173	143	287	257	476	537	439
125 000 to 129 999	150	80	133	205	439	422	343	152	124	196	178	380	407	338
130 000 to 134 999	154	101	222	176	483	346	354	126	61	185	204	423	414	313
135 000 to 139 999	114	113	148	184	357	277	332	111	111	183	173	366	362	313

© 2022 Avanceña ALV et al. *JAMA Health Forum.*

140 000 to 144 999	129	62	126	157	332	309	280	93	90	135	166	340	301	266
145 000 to 149 999	121	91	144	131	303	308	266	104	61	96	149	299	294	275
150 000 to 154 999	108	65	185	201	444	348	358	140	94	167	230	399	389	327
155 000 to 159 999	102	67	140	159	276	258	256	109	79	152	160	269	281	195
160 000 to 164 999	118	95	142	121	300	330	246	134	62	119	125	300	357	243
165 000 to 169 999	101	71	160	96	219	281	228	104	71	85	116	220	267	208
170 000 to 174 999	129	93	86	123	315	324	212	97	66	100	129	335	336	237
175 000 to 179 999	108	81	136	93	225	276	205	119	58	74	111	225	277	176
180 000 to 184 999	70	72	82	104	223	238	226	97	57	80	108	201	227	177
185 000 to 189 999	92	58	106	73	150	193	227	70	32	90	75	177	183	232
190 000 to 194 999	105	70	64	74	235	225	168	59	46	96	57	209	238	159
195 000 to 199 999	93	42	81	57	158	158	152	63	40	52	85	137	158	150
200 000 and over	1 205	801	1 344	1 276	3 028	3 455	3 294	1 140	790	922	1 194	3 140	3 452	2 937

© 2022 Avanceña ALV et al. *JAMA Health Forum.*

Since the population numbers are reported in multi-year age groups, we generated estimates of population numbers by age group using different assumptions. Under the “median age assumption,” we assumed that individuals in each age group had the median age of that group rounded to the nearest year (eTable 3). For example, in the 18-21-year age group, we assumed that all individuals were aged 20. In contrast, under the “equal distribution assumption,” we evenly divided the number of people in each age group across the ages in that group. For example, if there are 4 million 18-21-year-olds, we assumed that there are 1 million each of 18-, 19-, 20-, and 21-year-olds. We applied the same assumptions for both genders and all household income groups.

eTable 3. Median ages used in the median assumption

Age group (in years)	Median age
18-21	20
22-24	23
25-29	27
30-34	32
35-44	40
45-54	50
55-64	60

© 2022 Avanceña ALV et al. *JAMA Health Forum.*

C. Mortality rates

Systematic review

We conducted a narrative review with a systematic search approach of the literature on income and health to derive parameters for our simulation analysis. With the help of a health services informatician, we ran queries in PubMed, Scopus, and the National Bureau of Economic Research database to identify all studies (i.e. experimental studies, cohort studies, case-control studies, observational studies, etc.) investigating the relationship between income and/or wealth and life expectancy and mortality rates in the United States. The inclusion and exclusion criteria are listed in eTable 4. We first conducted title and abstract review of all yielded manuscripts (n = 554) to identify 176 manuscripts for full text review using DistillerSR. Next, two authors reviewed the full text of each of the articles which met our inclusion/exclusion criteria. Following full text review, we selected two studies and used the mortality rate ratio estimates from these studies to calculate age-, gender-, and household income group-specific all-cause mortality rates for our simulation model.

eTable 4. Inclusion and exclusion criteria

Inclusion criteria	Exclusion criteria
• Objective of study was to describe or measure the nonlinear relationship between income and health<br>• Measure of health used was all-cause mortality or life expectancy<br>• US-based study	• Measures of income are GDP or GNI<br>• Meta-analyses or reviews<br>• Measure of health used was disease-specific mortality<br>• Limited sample (i.e., only includes single gender, age group, race, etc.)

The first study we found used data from years 2008-2011 of the National Longitudinal Mortality Study (NLMS), which matches individuals in the ASEC to the National Death Index, to estimate the absolute effect of household income on mortality.[8] In multivariate regression models that used inflation-adjusted and equivalized annual household income as the covariate of interest, the study found that males in the lowest household income levels (≤$5 969 in 1990 US$) had mortality incident rate ratios equal to 2.23 (95% CI 2.04-2.43) when compared to males with average household incomes ($26 837-32 446 in 1990 US$). On the other hand, males in the highest household income group (≥$53 974) had a mortality incident rate ratio of 0.68 (95% CI 0.65-0.71) when compared to males with average household incomes. For females, the mortality disparities by household income were slightly larger than that for males.

The second study used data from the Panel Study of Income Dynamics (PSID), a longitudinal study of a representative sample of US individuals and their families that started in 1968, to characterize the relationship of total family income and mortality.[9] The authors found that individuals in 1990 (the most recent year in their analysis) who had household incomes below the 32nd percentile ($33 080) experienced a 54.7% reduction in mortality for every $10 000 increase in household income. At household incomes above this threshold, individuals experienced a more modest reduction (5.3%) in mortality per $10 000-increase in household income, and this finding was not statistically significant.

Estimation of base mortality rates

<u>NLMS</u>

The study that used data from NLMS reported the mortality incident rate ratios by gender for each household income group (adjusted to 1990 US$). We inflated these household income groups into 2019 US$ using the Consumer Price Index (CPI).[10] In order to calculate age-, gender-, and household income group-specific mortality rates, we first calculated the proportion of people in each household income group by age and gender using data from ASEC.[7] The ASEC reports the breakdown of people in households by total household income in $5 000 increments so we assumed that people were uniformly distributed within each $5 000 household income bracket. eTables 5 and 6 report the proportion of people in each NLMS household income group by age and gender.

Using the proportion of people in each household income group and the reported mortality incident rate ratios for each household income group, we calculated the age-, gender-, and household income group-specific mortality rates. Specifically, for each age x and gender g, we estimated the mortality rate for each household income group, q_i, by solving the following equality expressed in matrix form (Eq. 1):

© 2022 Avanceña ALV et al. *JAMA Health Forum.*

$$\begin{bmatrix} p_1 & p_2 & p_3 & p_4 & p_5 & p_6 & p_7 & p_8 & p_9 & p_{10} \\ -1 & 0 & 0 & 0 & 0 & 0 & r_1 & 0 & 0 & 0 \\ 0 & -1 & 0 & 0 & 0 & 0 & r_2 & 0 & 0 & 0 \\ 0 & 0 & -1 & 0 & 0 & 0 & r_3 & 0 & 0 & 0 \\ 0 & 0 & 0 & -1 & 0 & 0 & r_4 & 0 & 0 & 0 \\ 0 & 0 & 0 & 0 & -1 & 0 & r_5 & 0 & 0 & 0 \\ 0 & 0 & 0 & 0 & 0 & -1 & r_6 & 0 & 0 & 0 \\ 0 & 0 & 0 & 0 & 0 & 0 & r_8 & -1 & 0 & 0 \\ 0 & 0 & 0 & 0 & 0 & 0 & r_9 & 0 & -1 & 0 \\ 0 & 0 & 0 & 0 & 0 & 0 & r_{10} & 0 & 0 & -1 \end{bmatrix} \begin{bmatrix} q_1 \\ q_2 \\ q_3 \\ q_4 \\ q_5 \\ q_6 \\ q_7 \\ q_8 \\ q_9 \\ q_{10} \end{bmatrix} = \begin{bmatrix} z \\ 0 \\ 0 \\ 0 \\ 0 \\ 0 \\ 0 \\ 0 \\ 0 \\ 0 \end{bmatrix} \quad \text{(Eq. 1)}$$

where i indicates household income group, p_i is proportion of people in each household income group, r_i is mortality incident rate ratios by household income group (relative to the seventh household income group, which is the reference group), and z is the overall probability of death based on the CDC life table.[11] eTables 7 and 8 show base estimate of age-, gender-, and household income group-specific mortality rates using the NLMS, and Figure 1 in the main manuscript shows how the calculated household income-based, all-cause mortality rates among females differ from the CDC-reported all-cause mortality rates. The mortality rates were estimated using the statistical program R Version 4.0.5 (R Foundation for Statistical Computing, Vienna, Austria).

© 2022 Avanceña ALV et al. *JAMA Health Forum.*

eTable 5. Proportion of males in each National Longitudinal Mortality Study household income group by age and gender using data from the 2020 Current Population Survey[5]

Age	Household income group (2019 US$)									
	1 (≤$11 676)	2 ($11 677-19 915)	3 ($19 916-27 708)	4 ($27 709-35 348)	5 ($35 349-43 505)	6 ($43 506-52 493)	7 ($52 494-63 466)	8 ($63 467-78 567)	9 ($78 568-105 574)	10 (≥$105 575)
18	0.039326379	0.00143591	0.00124385	0.00112495	0.00104264	0.00097862	0.00091459	0.00085057	0.00078655	0.00062192
19	0.039326379	0.00167328	0.00144947	0.00131092	0.00121499	0.00114039	0.00106578	0.00099118	0.00091657	0.00072473
20	0.039326379	0.00191734	0.00166088	0.00150212	0.00139221	0.00130672	0.00122123	0.00113575	0.00105026	0.00083044
21	0.039326379	0.00214969	0.00186215	0.00168415	0.00156092	0.00146508	0.00136923	0.00127338	0.00117754	0.00093108
22	0.039335469	0.00231314	0.00200374	0.0018122	0.0016796	0.00157647	0.00147334	0.0013702	0.00126707	0.00100187
23	0.039335469	0.00245657	0.00212799	0.00192458	0.00178376	0.00167423	0.0015647	0.00145517	0.00134564	0.00106399
24	0.039335469	0.00256209	0.00221939	0.00200724	0.00186037	0.00174614	0.00163191	0.00151767	0.00140344	0.0011097
25	0.028958346	0.00270117	0.00233986	0.0021162	0.00196136	0.00184092	0.00172049	0.00160005	0.00147962	0.00116993
26	0.028958346	0.0027935	0.00241985	0.00218854	0.0020284	0.00190385	0.0017793	0.00165475	0.0015302	0.00120992
27	0.028958346	0.00287576	0.0024911	0.00225298	0.00208813	0.00195991	0.00183169	0.00170348	0.00157526	0.00124555
28	0.028958346	0.00295802	0.00256236	0.00231743	0.00214786	0.00201598	0.00188409	0.0017522	0.00162032	0.00128118
29	0.028958346	0.0030386	0.00263217	0.00238056	0.00220637	0.00207089	0.00193542	0.00179994	0.00166446	0.00131608
30	0.023696035	0.00317282	0.00274843	0.00248572	0.00230383	0.00216237	0.00202091	0.00187944	0.00173798	0.00137422
31	0.023696035	0.00325475	0.0028194	0.0025499	0.00236332	0.0022182	0.00207309	0.00192797	0.00178286	0.0014097
32	0.023696035	0.0033435	0.00289628	0.00261943	0.00242776	0.00227869	0.00212962	0.00198054	0.00183147	0.00144814
33	0.023696035	0.00343737	0.00297759	0.00269297	0.00249592	0.00234267	0.00218941	0.00203615	0.00188289	0.0014888
34	0.023696035	0.00353636	0.00306334	0.00277052	0.0025678	0.00241013	0.00225246	0.00209479	0.00193711	0.00153167
35	0.027018825	0.0037276	0.00322901	0.00292035	0.00270667	0.00254047	0.00237427	0.00220807	0.00204187	0.0016145
36	0.027018825	0.00385308	0.0033377	0.00301865	0.00279778	0.00262598	0.00245419	0.0022824	0.0021106	0.00166885
37	0.027018825	0.00396809	0.00343733	0.00310876	0.00288129	0.00270437	0.00252745	0.00235053	0.00217361	0.00171867
38	0.027018825	0.00406568	0.00352187	0.00318522	0.00295215	0.00277088	0.00258961	0.00240834	0.00222706	0.00176093
39	0.027018825	0.00416502	0.00360791	0.00326304	0.00302428	0.00283858	0.00265288	0.00246718	0.00228148	0.00180396
40	0.027018825	0.00429223	0.00371811	0.00336271	0.00311665	0.00292528	0.00273391	0.00254253	0.00235116	0.00185906
41	0.027018825	0.00447173	0.0038736	0.00350333	0.00324699	0.00304761	0.00284824	0.00264886	0.00244948	0.0019368
42	0.027018825	0.00470699	0.0040774	0.00368764	0.00341782	0.00320795	0.00299808	0.00278822	0.00257835	0.0020387
43	0.027018825	0.00500151	0.00433252	0.00391838	0.00363167	0.00340867	0.00318567	0.00296268	0.00273968	0.00216626
44	0.027018825	0.00534307	0.00462839	0.00418597	0.00387968	0.00364146	0.00340323	0.00316501	0.00292678	0.0023142

© 2022 Avanceña ALV et al. JAMA Health Forum.

45	0.028525946	0.00577226	0.00500018	0.00452222	0.00419132	0.00393396	0.0036766	0.00341924	0.00316188	0.00250009
46	0.028525946	0.00619462	0.00536604	0.00485311	0.00449801	0.00422181	0.00394562	0.00366943	0.00339323	0.00268302
47	0.028525946	0.00669442	0.00579898	0.00524467	0.00486091	0.00456244	0.00426396	0.00396548	0.003667	0.00289949
48	0.028525946	0.00729628	0.00632034	0.00571619	0.00529794	0.00497262	0.00464731	0.004322	0.00399669	0.00316017
49	0.028525946	0.00800197	0.00693165	0.00626906	0.00581035	0.00545357	0.0050968	0.00474002	0.00438325	0.00346582
50	0.028525946	0.00876046	0.00758868	0.00686329	0.0063611	0.00597051	0.00557991	0.00518932	0.00479872	0.00379434
51	0.028525946	0.00957527	0.0082945	0.00750164	0.00695274	0.00652582	0.0060989	0.00567197	0.00524505	0.00414725
52	0.028525946	0.01049742	0.00909331	0.00822409	0.00762233	0.00715429	0.00668625	0.00621822	0.00575018	0.00454665
53	0.028525946	0.01152516	0.00998358	0.00902927	0.00836859	0.00785473	0.00734087	0.00682701	0.00631315	0.00499179
54	0.028525946	0.01261802	0.01093026	0.00988546	0.00916213	0.00859954	0.00803696	0.00747437	0.00691178	0.00546513
55	0.043009827	0.01288147	0.01115847	0.01009185	0.00935343	0.00877909	0.00820476	0.00763043	0.00705609	0.00557924
56	0.043009827	0.01394131	0.01207655	0.01092217	0.01012299	0.0095014	0.00887981	0.00825823	0.00763664	0.00603827
57	0.043009827	0.01504902	0.01303609	0.01179	0.01092731	0.01025634	0.00958536	0.00891439	0.00824341	0.00651805
58	0.043009827	0.01624092	0.01406857	0.01272378	0.01179277	0.01106865	0.01034454	0.00962042	0.0088963	0.00703429
59	0.043009827	0.01753023	0.01518542	0.01373387	0.01272895	0.01194735	0.01116575	0.01038415	0.00960254	0.00759271
60	0.043009827	0.01893509	0.01640237	0.0148345	0.01374904	0.0129048	0.01206057	0.01121633	0.01037209	0.00820118
61	0.043009827	0.02040598	0.01767652	0.01598685	0.01481708	0.01390726	0.01299744	0.01208762	0.0111778	0.00883826
62	0.043009827	0.02189008	0.01896211	0.01714956	0.01589471	0.01491872	0.01394273	0.01296674	0.01199075	0.00948106
63	0.043009827	0.02334282	0.02022053	0.01828769	0.01694956	0.0159088	0.01486804	0.01382728	0.01278651	0.01011027
64	0.043009827	0.0247939	0.02147752	0.01942452	0.01800322	0.01689776	0.0157923	0.01468683	0.01358137	0.01073876

© 2022 Avanceña ALV et al. JAMA Health Forum.

eTable 6. Proportion of females in each National Longitudinal Mortality Study household income group by age and gender using data from the 2020 Current Population Survey[5]

Age	Household income (2019 $)									
	1 (≤$11 676)	2 ($11 677-19 915)	3 ($19 916-27 708)	4 ($27 709-35 348)	5 ($35 349-43 505)	6 ($43 506-52 493)	7 ($52 494-63 466)	8 ($63 467-78 567)	9 ($78 568-105 574)	10 (≥$105 575)
18	0.03818544	0.00143591	0.00124385	0.00112495	0.00104264	0.00097862	0.00091459	0.00085057	0.00078655	0.00062192
19	0.03818544	0.00167328	0.00144947	0.00131092	0.00121499	0.00114039	0.00106578	0.00099118	0.00091657	0.00072473
20	0.03818544	0.00191734	0.00166088	0.00150212	0.00139221	0.00130672	0.00122123	0.00113575	0.00105026	0.00083044
21	0.03818544	0.00214969	0.00186215	0.00168415	0.00156092	0.00146508	0.00136923	0.00127338	0.00117754	0.00093108
22	0.049690327	0.00231314	0.00200374	0.0018122	0.0016796	0.00157647	0.00147334	0.0013702	0.00126707	0.00100187
23	0.049690327	0.00245657	0.00212799	0.00192458	0.00178376	0.00167423	0.0015647	0.00145517	0.00134564	0.00106399
24	0.049690327	0.00256209	0.00221939	0.00200724	0.00186037	0.00174614	0.00163191	0.00151767	0.00140344	0.0011097
25	0.048588971	0.00270117	0.00233986	0.0021162	0.00196136	0.00184092	0.00172049	0.00160005	0.00147962	0.00116993
26	0.048588971	0.0027935	0.00241985	0.00218854	0.0020284	0.00190385	0.0017793	0.00165475	0.0015302	0.00120992
27	0.048588971	0.00287576	0.0024911	0.00225298	0.00208813	0.00195991	0.00183169	0.00170348	0.00157526	0.00124555
28	0.048588971	0.00295802	0.00256236	0.00231743	0.00214786	0.00201598	0.00188409	0.0017522	0.00162032	0.00128118
29	0.048588971	0.0030386	0.00263217	0.00238056	0.00220637	0.00207089	0.00193542	0.00179994	0.00166446	0.00131608
30	0.036138419	0.00317282	0.00274843	0.00248572	0.00230383	0.00216237	0.00202091	0.00187944	0.00173798	0.00137422
31	0.036138419	0.00325475	0.0028194	0.0025499	0.00236332	0.0022182	0.00207309	0.00192797	0.00178286	0.0014097
32	0.036138419	0.0033435	0.00289628	0.00261943	0.00242776	0.00227869	0.00212962	0.00198054	0.00183147	0.00144814
33	0.036138419	0.00343737	0.00297759	0.00269297	0.00249592	0.00234267	0.00218941	0.00203615	0.00188289	0.0014888
34	0.036138419	0.00353636	0.00306334	0.00277052	0.0025678	0.00241013	0.00225246	0.00209479	0.00193711	0.00153167
35	0.030538715	0.0037276	0.00322901	0.00292035	0.00270667	0.00254047	0.00237427	0.00220807	0.00204187	0.0016145
36	0.030538715	0.00385308	0.0033377	0.00301865	0.00279778	0.00262598	0.00245419	0.0022824	0.0021106	0.00166885
37	0.030538715	0.00396809	0.00343733	0.00310876	0.00288129	0.00270437	0.00252745	0.00235053	0.00217361	0.00171867
38	0.030538715	0.00406568	0.00352187	0.00318522	0.00295215	0.00277088	0.00258961	0.00240834	0.00222706	0.00176093
39	0.030538715	0.00416502	0.00360791	0.00326304	0.00302428	0.00283858	0.00265288	0.00246718	0.00228148	0.00180396
40	0.030538715	0.00429223	0.00371811	0.00336271	0.00311665	0.00292528	0.00273391	0.00254253	0.00235116	0.00185906
41	0.030538715	0.00447173	0.0038736	0.00350333	0.00324699	0.00304761	0.00284824	0.00264886	0.00244948	0.0019368
42	0.030538715	0.00470699	0.0040774	0.00368764	0.00341782	0.00320795	0.00299808	0.00278822	0.00257835	0.0020387
43	0.030538715	0.00500151	0.00433252	0.00391838	0.00363167	0.00340867	0.00318567	0.00296268	0.00273968	0.00216626
44	0.030538715	0.00534307	0.00462839	0.00418597	0.00387968	0.00364146	0.00340323	0.00316501	0.00292678	0.0023142

© 2022 Avanceña ALV et al. JAMA Health Forum.

45	0.036376015	0.00577226	0.00500018	0.00452222	0.00419132	0.00393396	0.0036766	0.00341924	0.00316188	0.00250009
46	0.036376015	0.00619462	0.00536604	0.00485311	0.00449801	0.00422181	0.00394562	0.00366943	0.00339323	0.00268302
47	0.036376015	0.00669442	0.00579898	0.00524467	0.00486091	0.00456244	0.00426396	0.00396548	0.003667	0.00289949
48	0.036376015	0.00729628	0.00632034	0.00571619	0.00529794	0.00497262	0.00464731	0.004322	0.00399669	0.00316017
49	0.036376015	0.00800197	0.00693165	0.00626906	0.00581035	0.00545357	0.0050968	0.00474002	0.00438325	0.00346582
50	0.036376015	0.00876046	0.00758868	0.00686329	0.0063611	0.00597051	0.00557991	0.00518932	0.00479872	0.00379434
51	0.036376015	0.00957527	0.0082945	0.00750164	0.00695274	0.00652582	0.0060989	0.00567197	0.00524505	0.00414725
52	0.036376015	0.01049742	0.00909331	0.00822409	0.00762233	0.00715429	0.00668625	0.00621822	0.00575018	0.00454665
53	0.036376015	0.01152516	0.00998358	0.00902927	0.00836859	0.00785473	0.00734087	0.00682701	0.00631315	0.00499179
54	0.036376015	0.01261802	0.01093026	0.00988546	0.00916213	0.00859954	0.00803696	0.00747437	0.00691178	0.00546513
55	0.059378708	0.01288147	0.01115847	0.01009185	0.00935343	0.00877909	0.00820476	0.00763043	0.00705609	0.00557924
56	0.059378708	0.01394131	0.01207655	0.01092217	0.01012299	0.0095014	0.00887981	0.00825823	0.00763664	0.00603827
57	0.059378708	0.01504902	0.01303609	0.01179	0.01092731	0.01025634	0.00958536	0.00891439	0.00824341	0.00651805
58	0.059378708	0.01624092	0.01406857	0.01272378	0.01179277	0.01106865	0.01034454	0.00962042	0.0088963	0.00703429
59	0.059378708	0.01753023	0.01518542	0.01373387	0.01272895	0.01194735	0.01116575	0.01038415	0.00960254	0.00759271
60	0.059378708	0.01893509	0.01640237	0.0148345	0.01374904	0.0129048	0.01206057	0.01121633	0.01037209	0.00820118
61	0.059378708	0.02040598	0.01767652	0.01598685	0.01481708	0.01390726	0.01299744	0.01208762	0.0111778	0.00883826
62	0.059378708	0.02189008	0.01896211	0.01714956	0.01589471	0.01491872	0.01394273	0.01296674	0.01199075	0.00948106
63	0.059378708	0.02334282	0.02022053	0.01828769	0.01694956	0.0159088	0.01486804	0.01382728	0.01278651	0.01011027
64	0.059378708	0.0247939	0.02147752	0.01942452	0.01800322	0.01689776	0.0157923	0.01468683	0.01358137	0.01073876

© 2022 Avanceña ALV et al. JAMA Health Forum.

eTable 7. Base estimate of age- and household income group-specific mortality rates among males estimated using data from National Longitudinal Mortality Study[8]

Age	Household income group (2019 US$)									
	1 (≤$11 676)	2 ($11 677-19 915)	3 ($19 916-27 708)	4 ($27 709-35 348)	5 ($35 349-43 505)	6 ($43 506-52 493)	7 ($52 494-63 466)	8 ($63 467-78 567)	9 ($78 568-105 574)	10 (≥$105 575)
18	0.00203955	0.00143591	0.00124385	0.00112495	0.00104264	0.00097862	0.00091459	0.00085057	0.00078655	0.00062192
19	0.0023767	0.00167328	0.00144947	0.00131092	0.00121499	0.00114039	0.00106578	0.00099118	0.00091657	0.00072473
20	0.00272335	0.00191734	0.00166088	0.00150212	0.00139221	0.00130672	0.00122123	0.00113575	0.00105026	0.00083044
21	0.00305338	0.00214969	0.00186215	0.00168415	0.00156092	0.00146508	0.00136923	0.00127338	0.00117754	0.00093108
22	0.00328554	0.00231314	0.00200374	0.0018122	0.0016796	0.00157647	0.00147334	0.0013702	0.00126707	0.00100187
23	0.00348927	0.00245657	0.00212799	0.00192458	0.00178376	0.00167423	0.0015647	0.00145517	0.00134564	0.00106399
24	0.00363915	0.00256209	0.00221939	0.00200724	0.00186037	0.00174614	0.00163191	0.00151767	0.00140344	0.0011097
25	0.00383669	0.00270117	0.00233986	0.0021162	0.00196136	0.00184092	0.00172049	0.00160005	0.00147962	0.00116993
26	0.00396784	0.0027935	0.00241985	0.00218854	0.0020284	0.00190385	0.0017793	0.00165475	0.0015302	0.00120992
27	0.00408468	0.00287576	0.0024911	0.00225298	0.00208813	0.00195991	0.00183169	0.00170348	0.00157526	0.00124555
28	0.00420152	0.00295802	0.00256236	0.00231743	0.00214786	0.00201598	0.00188409	0.0017522	0.00162032	0.00128118
29	0.00431598	0.0030386	0.00263217	0.00238056	0.00220637	0.00207089	0.00193542	0.00179994	0.00166446	0.00131608
30	0.00450662	0.00317282	0.00274843	0.00248572	0.00230383	0.00216237	0.00202091	0.00187944	0.00173798	0.00137422
31	0.00462299	0.00325475	0.0028194	0.0025499	0.00236332	0.0022182	0.00207309	0.00192797	0.00178286	0.0014097
32	0.00474904	0.0033435	0.00289628	0.00261943	0.00242776	0.00227869	0.00212962	0.00198054	0.00183147	0.00144814
33	0.00488238	0.00343737	0.00297759	0.00269297	0.00249592	0.00234267	0.00218941	0.00203615	0.00188289	0.0014888
34	0.00502298	0.00353636	0.00306334	0.00277052	0.0025678	0.00241013	0.00225246	0.00209479	0.00193711	0.00153167
35	0.00529462	0.0037276	0.00322901	0.00292035	0.00270667	0.00254047	0.00237427	0.00220807	0.00204187	0.0016145
36	0.00547284	0.00385308	0.0033377	0.00301865	0.00279778	0.00262598	0.00245419	0.0022824	0.0021106	0.00166885
37	0.00563621	0.00396809	0.00343733	0.00310876	0.00288129	0.00270437	0.00252745	0.00235053	0.00217361	0.00171867
38	0.00577483	0.00406568	0.00352187	0.00318522	0.00295215	0.00277088	0.00258961	0.00240834	0.00222706	0.00176093
39	0.00591592	0.00416502	0.00360791	0.00326304	0.00302428	0.00283858	0.00265288	0.00246718	0.00228148	0.00180396
40	0.00609661	0.00429223	0.00371811	0.00336271	0.00311665	0.00292528	0.00273391	0.00254253	0.00235116	0.00185906
41	0.00635157	0.00447173	0.0038736	0.00350333	0.00324699	0.00304761	0.00284824	0.00264886	0.00244948	0.0019368
42	0.00668573	0.00470699	0.0040774	0.00368764	0.00341782	0.00320795	0.00299808	0.00278822	0.00257835	0.0020387
43	0.00710405	0.00500151	0.00433252	0.00391838	0.00363167	0.00340867	0.00318567	0.00296268	0.00273968	0.00216626
44	0.00758921	0.00534307	0.00462839	0.00418597	0.00387968	0.00364146	0.00340323	0.00316501	0.00292678	0.0023142
45	0.00819882	0.00577226	0.00500018	0.00452222	0.00419132	0.00393396	0.0036766	0.00341924	0.00316188	0.00250009

© 2022 Avanceña ALV et al. JAMA Health Forum.

46	0.00879873	0.00619462	0.00536604	0.00485311	0.00449801	0.00422181	0.00394562	0.00366943	0.00339323	0.00268302
47	0.00950863	0.00669442	0.00579898	0.00524467	0.00486091	0.00456244	0.00426396	0.00396548	0.003667	0.00289949
48	0.0103635	0.00729628	0.00632034	0.00571619	0.00529794	0.00497262	0.00464731	0.004322	0.00399669	0.00316017
49	0.01136586	0.00800197	0.00693165	0.00626906	0.00581035	0.00545357	0.0050968	0.00474002	0.00438325	0.00346582
50	0.0124432	0.00876046	0.00758868	0.00686329	0.0063611	0.00597051	0.00557991	0.00518932	0.00479872	0.00379434
51	0.01360054	0.00957527	0.0082945	0.00750164	0.00695274	0.00652582	0.0060989	0.00567197	0.00524505	0.00414725
52	0.01491035	0.01049742	0.00909331	0.00822409	0.00762233	0.00715429	0.00668625	0.00621822	0.00575018	0.00454665
53	0.01637014	0.01152516	0.00998358	0.00902927	0.00836859	0.00785473	0.00734087	0.00682701	0.00631315	0.00499179
54	0.01792241	0.01261802	0.01093026	0.00988546	0.00916213	0.00859954	0.00803696	0.00747437	0.00691178	0.00546513
55	0.01829661	0.01288147	0.01115847	0.01009185	0.00935343	0.00877909	0.00820476	0.00763043	0.00705609	0.00557924
56	0.01980199	0.01394131	0.01207655	0.01092217	0.01012299	0.0095014	0.00887981	0.00825823	0.00763664	0.00603827
57	0.02137536	0.01504902	0.01303609	0.01179	0.01092731	0.01025634	0.00958536	0.00891439	0.00824341	0.00651805
58	0.02306832	0.01624092	0.01406857	0.01272378	0.01179277	0.01106865	0.01034454	0.00962042	0.0088963	0.00703429
59	0.02489962	0.01753023	0.01518542	0.01373387	0.01272895	0.01194735	0.01116575	0.01038415	0.00960254	0.00759271
60	0.02689506	0.01893509	0.01640237	0.0148345	0.01374904	0.0129048	0.01206057	0.01121633	0.01037209	0.00820118
61	0.02898429	0.02040598	0.01767652	0.01598685	0.01481708	0.01390726	0.01299744	0.01208762	0.0111778	0.00883826
62	0.03109228	0.02189008	0.01896211	0.01714956	0.01589471	0.01491872	0.01394273	0.01296674	0.01199075	0.00948106
63	0.03315572	0.02334282	0.02022053	0.01828769	0.01694956	0.0159088	0.01486804	0.01382728	0.01278651	0.01011027
64	0.03521682	0.0247939	0.02147752	0.01942452	0.01800322	0.01689776	0.0157923	0.01468683	0.01358137	0.01073876

© 2022 Avanceña ALV et al. JAMA Health Forum.

eTable 8. Base estimate of age- and household income group-specific mortality rates among females estimated using data from National Longitudinal Mortality Study[8]

Age	Household income group (2019 US$)									
	1 (≤$11 676)	2 ($11 677-19 915)	3 ($19 916-27 708)	4 ($27 709-35 348)	5 ($35 349-43 505)	6 ($43 506-52 493)	7 ($52 494-63 466)	8 ($63 467-78 567)	9 ($78 568-105 574)	10 (≥$105 575)
18	0.000823275	0.000567776	0.000489707	0.000440026	0.000408089	0.0003797	0.00035486	0.00033002	0.000301631	0.000237756
19	0.000928634	0.000640438	0.000552377	0.000496339	0.000460314	0.000428293	0.000400273	0.000372254	0.000340232	0.000268183
20	0.001038894	0.000716479	0.000617963	0.000555271	0.000514969	0.000479145	0.000447799	0.000416453	0.000380629	0.000300026
21	0.001154055	0.0007959	0.000686464	0.000616822	0.000572053	0.000532258	0.000497437	0.000462617	0.000422822	0.000333283
22	0.001197616	0.000825942	0.000712375	0.000640105	0.000593646	0.000552349	0.000516214	0.000480079	0.000438782	0.000345863
23	0.001283993	0.000885513	0.000763755	0.000686272	0.000636462	0.000592187	0.000553445	0.000514704	0.000470429	0.000370808
24	0.001361033	0.000938643	0.00080958	0.000727449	0.00067465	0.000627718	0.000586652	0.000545587	0.000498654	0.000393057
25	0.001443872	0.000995774	0.000858855	0.000771725	0.000715712	0.000665924	0.000622359	0.000578794	0.000529005	0.00041698
26	0.001521601	0.00104938	0.00090509	0.000813269	0.000754242	0.000701773	0.000655862	0.000609952	0.000557483	0.000439428
27	0.001606396	0.001107859	0.000955529	0.000858591	0.000796274	0.000740881	0.000692412	0.000643943	0.00058855	0.000463916
28	0.001705324	0.001176085	0.001014374	0.000911466	0.000845311	0.000786507	0.000735053	0.0006836	0.000624795	0.000492486
29	0.001823095	0.001257307	0.001084427	0.000974413	0.000903689	0.000840824	0.000785817	0.000730809	0.000667944	0.000526497
30	0.002024395	0.001396135	0.001204166	0.001082004	0.001003472	0.000933665	0.000872584	0.000811503	0.000741697	0.000584631
31	0.002163756	0.001492245	0.001287062	0.00115649	0.001072551	0.000997939	0.000932653	0.000867368	0.000792755	0.000624878
32	0.002298226	0.001584984	0.001367048	0.001228362	0.001139207	0.001059958	0.000990615	0.000921272	0.000842023	0.000663712
33	0.002418028	0.001667605	0.001438309	0.001292394	0.001198591	0.001115211	0.001042253	0.000969296	0.000885915	0.00069831
34	0.002532939	0.001746854	0.001506662	0.001353812	0.001255552	0.001168209	0.001091784	0.001015359	0.000928016	0.000731495
35	0.002739308	0.001889178	0.001629416	0.001464113	0.001357846	0.001263388	0.001180736	0.001098084	0.001003626	0.000791093
36	0.002882951	0.001988242	0.001714859	0.001540888	0.001429049	0.001329637	0.001242651	0.001155666	0.001056254	0.000832576
37	0.003031635	0.002090783	0.0018033	0.001620357	0.00150275	0.001398211	0.001306739	0.001215267	0.001110728	0.000875515
38	0.003185359	0.002196799	0.001894739	0.001702519	0.001578949	0.001469109	0.001372999	0.001276889	0.001167049	0.00091991
39	0.003356723	0.002314981	0.001996671	0.00179411	0.001663893	0.001548144	0.001446863	0.001345583	0.001229834	0.000969398
40	0.003563368	0.002457495	0.00211959	0.001904559	0.001766325	0.00164345	0.001535934	0.001428419	0.001305544	0.001029076
41	0.003812854	0.002629555	0.002267991	0.002037905	0.001889992	0.001758515	0.001643472	0.001528429	0.001396951	0.001101126
42	0.004097621	0.002825946	0.002437378	0.002190108	0.002031148	0.001889851	0.001766216	0.001642581	0.001501284	0.001183365
43	0.004410109	0.003041454	0.002623254	0.002357127	0.002186045	0.002033973	0.001900909	0.001767845	0.001615773	0.001273609
44	0.004745277	0.003272605	0.002822622	0.002536269	0.002352185	0.002188554	0.002045378	0.001902202	0.001738571	0.001370403
45	0.00510983	0.003524021	0.003039468	0.002731116	0.00253289	0.002356689	0.002202513	0.002048337	0.001872136	0.001475684

© 2022 Avanceña ALV et al. JAMA Health Forum.

46	0.005508523	0.003798981	0.003276621	0.00294421	0.002730518	0.002540569	0.002374363	0.002208158	0.002018209	0.001590823
47	0.0059703	0.004117448	0.003551299	0.003191022	0.002959416	0.002753544	0.002573405	0.002393267	0.002187394	0.001724181
48	0.006520395	0.004496824	0.003878511	0.003485039	0.003232092	0.003007251	0.002810515	0.002613779	0.002388938	0.001883045
49	0.007156285	0.004935369	0.004256756	0.003824911	0.003547297	0.003300528	0.003084606	0.002868683	0.002621915	0.002066686
50	0.007835072	0.005403498	0.004660517	0.004187711	0.003883764	0.003613589	0.003377186	0.003140783	0.002870608	0.002262715
51	0.008556757	0.005901212	0.005089795	0.004573439	0.004241496	0.003946435	0.003688257	0.003430079	0.003135019	0.002471132
52	0.009364236	0.006458094	0.005570106	0.005005023	0.004641755	0.00431885	0.004036309	0.003753767	0.003430862	0.002704327
53	0.010260034	0.007075885	0.006102951	0.005483811	0.005085793	0.004731998	0.004422428	0.004112858	0.003759064	0.002963027
54	0.011206298	0.007728482	0.006665815	0.005989573	0.005554846	0.005168422	0.004830301	0.00449218	0.004105756	0.003236302
55	0.011208066	0.007729701	0.006666867	0.005990518	0.005555723	0.005169238	0.004831063	0.004492889	0.004106404	0.003236812
56	0.012117895	0.008357169	0.007208058	0.006476806	0.006006715	0.005588857	0.005223231	0.004857604	0.004439746	0.003499565
57	0.013027724	0.008984637	0.007749249	0.006963094	0.006457708	0.006008476	0.005615398	0.00522232	0.004773088	0.003762317
58	0.013951478	0.009621709	0.008298724	0.007456824	0.006915603	0.006434518	0.006013568	0.005592618	0.005111533	0.004029091
59	0.014921652	0.010290795	0.00887581	0.007975366	0.007396509	0.006881969	0.006431747	0.005981524	0.005466985	0.00430927
60	0.015968419	0.011012703	0.009498456	0.008534845	0.00791538	0.007364745	0.006882939	0.006401134	0.005850498	0.004611569
61	0.017108026	0.011798639	0.010176326	0.009143945	0.008480272	0.00789034	0.007374149	0.006857959	0.006268027	0.00494068
62	0.018342793	0.012650202	0.0109108	0.009803907	0.009092333	0.008459823	0.007906376	0.00735293	0.00672042	0.005297272
63	0.019684326	0.013575397	0.01170878	0.010520933	0.009757317	0.009078547	0.008484623	0.0078907	0.00721193	0.005684698
64	0.021146551	0.014583828	0.012578552	0.011302467	0.010482126	0.009752935	0.009114893	0.00847685	0.007747659	0.006106978

© 2022 Avanceña ALV et al. JAMA Health Forum.

PSID

To calculate age-, gender-, and household income group-specific mortality rates using the PSID, we first inflated costs from the PSID to 2019 US$ using the CPI[10]; thus, the minimum unit of household income that is associated with a decline in mortality risk was set at $14 897 (2019 US$) instead of the original $10 000 (2000 US$) reported in the study.[9] We then used the equation (Eq. 2):

$$q_i = q_0 * ((1-\Delta)^{\varphi/14897}) \text{ (Eq. 2)}$$

to estimate the risk of mortality at any household income level (q_i), where q_0 is baseline mortality, Δ is the change in mortality risk for each unit of household income gain estimated through the PSID, and φ is a person's annual household income. Baseline mortality (q_0), which represents the level of mortality risk that people in each age group experience regardless of household income, was estimated using the following formula (Eq. 3):

$$q_0 = \frac{z}{(\sum_1^{ik} p_{ik} * ((1-\Delta)^{\frac{\varphi_{ik}}{14\,897}}) + (p_k * (1-\Delta)^{49\,122/14\,897}))} \text{ (Eq. 3)}$$

where z is the overall probability of death at a certain age based on 2017 CDC life tables, p_{ik} is the proportion of the population in each household income group below the knot (eTables 5 and 6), φ_{ik} is the median household income in each household income group, and p_k is the proportion of the population with household incomes above the knot ($49 122 in 2019 US$), which is the household income threshold where any additional household income has no significant effect on mortality based on the PSID study. (A knot is a point that joins piecewise polynomial functions like linear splines.) Thus, we assumed individuals in the household income group that included the knot experienced mortalities equal to the sum of group mortalities above and below the knot, weighted by the proportion of that group's population above and below the knot. For our base-case analysis, Δ was set to 0.547. eTables 9 and 10 show the base estimates age-, gender-, and household income group-specific mortality rates using the PSID. All analyses were done in Microsoft Excel (Microsoft Corp, Redmond, Washington, USA).

eTable 9. Base estimate of age- and household income group-specific mortality rates among males estimated using data from Panel Study of Income Dynamics[9]

	**Household income group (2019 US$)**									
**Age**	**1 (≤$11 676)**	**2 ($11 677-19 915)**	**3 ($19 916-27 708)**	**4 ($27 709-35 348)**	**5 ($35 349-43 505)**	**6 ($43 506-52 493)**	**7 ($52 494-63 466)**	**8 ($63 467-78 567)**	**9 ($78 568-105 574)**	**10 (≥$105 575)**
18	0.004952607	0.002917044	0.00190597	0.001264656	0.00083105	0.000546413	0.00049663	0.00049663	0.00049663	0.00049663
19	0.005776826	0.003402502	0.002223164	0.001475122	0.000969355	0.000637348	0.00057928	0.00057928	0.00057928	0.00057928
20	0.006616593	0.003897118	0.002546341	0.001689558	0.001110268	0.000729998	0.000663488	0.000663488	0.000663488	0.000663488
21	0.007417637	0.004368926	0.002854616	0.001894106	0.001244684	0.000818376	0.000743814	0.000743814	0.000743814	0.000743814
22	0.00802822	0.004728554	0.003089594	0.002050019	0.00134714	0.00088574	0.000805041	0.000805041	0.000805041	0.000805041
23	0.008527027	0.005022347	0.003281556	0.002177391	0.00143084	0.000940773	0.00085506	0.00085506	0.00085506	0.00085506
24	0.00889229	0.005237484	0.003422125	0.002270661	0.001492132	0.000981072	0.000891687	0.000891687	0.000891687	0.000891687
25	0.01003188	0.005908693	0.003860686	0.002561657	0.001683356	0.001106801	0.001005961	0.001005961	0.001005961	0.001005961
26	0.01037144	0.006108691	0.003991363	0.002648364	0.001740334	0.001144264	0.001040011	0.001040011	0.001040011	0.001040011
27	0.010680901	0.006290961	0.004110457	0.002727386	0.001792262	0.001178406	0.001071043	0.001071043	0.001071043	0.001071043
28	0.010981677	0.006468116	0.004226208	0.00280419	0.001842732	0.00121159	0.001101204	0.001101204	0.001101204	0.001101204
29	0.011285651	0.006647154	0.00434319	0.00288181	0.001893739	0.001245127	0.001131685	0.001131685	0.001131685	0.001131685
30	0.012113966	0.007135025	0.00466196	0.003093322	0.002032731	0.001336514	0.001214745	0.001214745	0.001214745	0.001214745

© 2022 Avanceña ALV et al. JAMA Health Forum.

31	0.012431354	0.007321964	0.004784105	0.003174367	0.002085989	0.001371531	0.001246572	0.001246572	0.001246572	0.001246572
32	0.012768553	0.007520571	0.004913873	0.003260471	0.002142571	0.001408733	0.001280385	0.001280385	0.001280385	0.001280385
33	0.013126556	0.007731432	0.005051647	0.003351888	0.002202644	0.001448231	0.001316284	0.001316284	0.001316284	0.001316284
34	0.013501283	0.007952142	0.005195858	0.003447575	0.002265524	0.001489574	0.001353861	0.001353861	0.001353861	0.001353861
35	0.013864442	0.00816604	0.005335616	0.003540308	0.002326462	0.001529641	0.001390277	0.001390277	0.001390277	0.001390277
36	0.01433319	0.008442129	0.00551601	0.003660004	0.002405118	0.001581357	0.001437281	0.001437281	0.001437281	0.001437281
37	0.014757618	0.008692114	0.005679348	0.003768382	0.002476337	0.001628184	0.001479841	0.001479841	0.001479841	0.001479841
38	0.015121347	0.008906347	0.005819326	0.003861261	0.002537371	0.001668313	0.001516315	0.001516315	0.001516315	0.001516315
39	0.015490186	0.00912359	0.005961271	0.003955445	0.002599263	0.001709007	0.001553301	0.001553301	0.001553301	0.001553301
40	0.015962119	0.009401555	0.00614289	0.004075954	0.002678453	0.001761074	0.001600625	0.001600625	0.001600625	0.001600625
41	0.016628942	0.009794308	0.006399512	0.004246228	0.002790347	0.001834644	0.001667491	0.001667491	0.001667491	0.001667491
42	0.017506919	0.010311428	0.006737394	0.004470421	0.002937672	0.001931509	0.001755532	0.001755532	0.001755532	0.001755532
43	0.018600474	0.010955523	0.007158239	0.004749662	0.003121171	0.00205216	0.001865189	0.001865189	0.001865189	0.001865189
44	0.019873199	0.011705148	0.007648037	0.005074655	0.003334735	0.002192577	0.001992814	0.001992814	0.001992814	0.001992814
45	0.021091698	0.012422833	0.008116966	0.0053858	0.0035392	0.002327012	0.002115	0.002115	0.002115	0.002115
46	0.022634883	0.013331756	0.008710848	0.005779855	0.003798147	0.002497269	0.002269746	0.002269746	0.002269746	0.002269746
47	0.024459878	0.014406663	0.009413183	0.006245871	0.004104383	0.002698618	0.00245275	0.00245275	0.00245275	0.00245275
48	0.026659353	0.015702136	0.010259633	0.00680751	0.004473456	0.002941282	0.002673305	0.002673305	0.002673305	0.002673305
49	0.029237279	0.017220513	0.011251726	0.007465788	0.004906033	0.003225701	0.00293181	0.00293181	0.00293181	0.00293181
50	0.032011322	0.0188544	0.012319294	0.008174145	0.005371519	0.003531756	0.003209981	0.003209981	0.003209981	0.003209981
51	0.034986146	0.020606547	0.01346413	0.008933771	0.005870697	0.003859964	0.003508286	0.003508286	0.003508286	0.003508286
52	0.03835662	0.022591728	0.01476123	0.009794427	0.006436264	0.004231823	0.003846265	0.003846265	0.003846265	0.003846265
53	0.042108188	0.02480137	0.01620499	0.010752396	0.00706578	0.004645727	0.004222459	0.004222459	0.004222459	0.004222459
54	0.046103851	0.027154782	0.017742688	0.011772695	0.007736254	0.005086562	0.00462313	0.00462313	0.00462313	0.00462313
55	0.042511504	0.02503892	0.016360203	0.010855384	0.007133457	0.004690224	0.004262902	0.004262902	0.004262902	0.004262902
56	0.046006274	0.02709731	0.017705136	0.011747779	0.007719881	0.005075796	0.004613346	0.004613346	0.004613346	0.004613346
57	0.049664699	0.029252092	0.019113051	0.012681964	0.008333767	0.005479424	0.004980199	0.004980199	0.004980199	0.004980199
58	0.053597608	0.031568542	0.020626599	0.013686239	0.008993712	0.005913336	0.005374578	0.005374578	0.005374578	0.005374578
59	0.057853755	0.034075377	0.022264542	0.014773053	0.009707896	0.006382909	0.005801369	0.005801369	0.005801369	0.005801369
60	0.062487729	0.036804749	0.024047888	0.015956346	0.010485479	0.006894168	0.006266047	0.006266047	0.006266047	0.006266047
61	0.067343966	0.039665032	0.025916771	0.017196394	0.011300359	0.007429949	0.006753013	0.006753013	0.006753013	0.006753013
62	0.072241945	0.042549901	0.027801718	0.018447102	0.012122243	0.007970335	0.007244165	0.007244165	0.007244165	0.007244165

© 2022 Avanceña ALV et al. JAMA Health Forum.

63	0.077032867	0.045371714	0.029645465	0.019670471	0.012926162	0.008498909	0.007724582	0.007724582	0.007724582	0.007724582
64	0.081822338	0.048192672	0.031488654	0.020893471	0.013729839	0.009027323	0.008204853	0.008204853	0.008204853	0.008204853

© 2022 Avanceña ALV et al. JAMA Health Forum.

eTable 10. Base estimate of age- and household income group-specific mortality rates among females estimated using data from Panel Study of Income Dynamics[9]

	Household income group (2019 US$)									
**Age**	**1 (≤$11 676)**	**2 ($11 677-19 915)**	**3 ($19 916-27 708)**	**4 ($27 709-35 348)**	**5 ($35 349-43 505)**	**6 ($43 506-52 493)**	**7 ($52 494-63 466)**	**8 ($63 467-78 567)**	**9 ($78 568-105 574)**	**10 (≥$105 575)**
18	0.001892343	0.001114574	0.000728253	0.000483213	0.000317536	0.000208779	0.000189757	0.000189757	0.000189757	0.000189757
19	0.002136861	0.001258593	0.000822353	0.000545651	0.000358567	0.000235756	0.000214277	0.000214277	0.000214277	0.000214277
20	0.002392899	0.001409397	0.000920887	0.000611031	0.00040153	0.000264005	0.000239951	0.000239951	0.000239951	0.000239951
21	0.002652934	0.001562556	0.00102096	0.000677431	0.000445164	0.000292694	0.000266027	0.000266027	0.000266027	0.000266027
22	0.002667287	0.00157101	0.001026483	0.000681096	0.000447572	0.000294277	0.000267466	0.000267466	0.000267466	0.000267466
23	0.002859035	0.001683948	0.001100276	0.000730059	0.000479748	0.000315433	0.000286694	0.000286694	0.000286694	0.000286694
24	0.003028996	0.001784053	0.001165684	0.000773459	0.000508267	0.000334184	0.000303737	0.000303737	0.000303737	0.000303737
25	0.003295982	0.001941306	0.001268432	0.000841635	0.000553068	0.00036364	0.000330509	0.000330509	0.000330509	0.000330509
26	0.003469145	0.002043298	0.001335072	0.000885852	0.000582125	0.000382745	0.000347874	0.000347874	0.000347874	0.000347874
27	0.00366279	0.002157352	0.001409595	0.000935299	0.000614618	0.00040411	0.000367292	0.000367292	0.000367292	0.000367292
28	0.003892546	0.002292677	0.001498014	0.000993968	0.000653172	0.000429458	0.000390331	0.000390331	0.000390331	0.000390331
29	0.004157276	0.002448601	0.001599894	0.001061567	0.000697594	0.000458665	0.000416877	0.000416877	0.000416877	0.000416877
30	0.004865311	0.002865628	0.001872375	0.001242365	0.000816402	0.000536782	0.000487876	0.000487876	0.000487876	0.000487876
31	0.005195791	0.003060277	0.001999557	0.001326754	0.000871857	0.000573243	0.000521015	0.000521015	0.000521015	0.000521015
32	0.005517461	0.003249738	0.002123349	0.001408893	0.000925833	0.000608732	0.000553271	0.000553271	0.000553271	0.000553271
33	0.005809509	0.003421752	0.002235742	0.001483468	0.000974839	0.000640953	0.000582557	0.000582557	0.000582557	0.000582557
34	0.006081474	0.003581937	0.002340405	0.001552914	0.001020475	0.000670959	0.000609829	0.000609829	0.000609829	0.000609829
35	0.00662777	0.003903701	0.002550643	0.001692412	0.001112144	0.000731231	0.000664609	0.000664609	0.000664609	0.000664609
36	0.006978789	0.004110448	0.002685729	0.001782045	0.001171045	0.000769958	0.000699808	0.000699808	0.000699808	0.000699808
37	0.00733694	0.004321396	0.002823561	0.0018735	0.001231143	0.000809472	0.000735722	0.000735722	0.000735722	0.000735722
38	0.007709934	0.004541087	0.002967105	0.001968745	0.001293732	0.000850624	0.000773125	0.000773125	0.000773125	0.000773125
39	0.008126713	0.004786566	0.003127499	0.00207517	0.001363667	0.000896607	0.000814918	0.000814918	0.000814918	0.000814918
40	0.008625886	0.005080575	0.003319601	0.002202634	0.001447429	0.00095168	0.000864973	0.000864973	0.000864973	0.000864973
41	0.009228255	0.005435365	0.003551418	0.00235645	0.001548507	0.001018138	0.000925377	0.000925377	0.000925377	0.000925377
42	0.009919427	0.005842459	0.00381741	0.002532942	0.001664486	0.001094394	0.000994685	0.000994685	0.000994685	0.000994685
43	0.01067578	0.006287945	0.004108486	0.002726078	0.001791402	0.001177841	0.001070529	0.001070529	0.001070529	0.001070529
44	0.01148706	0.006765782	0.004420701	0.00293324	0.001927536	0.001267348	0.001151882	0.001151882	0.001151882	0.001151882
45	0.011926558	0.007024643	0.004589838	0.003045466	0.002001284	0.001315837	0.001195953	0.001195953	0.001195953	0.001195953

© 2022 Avanceña ALV et al. JAMA Health Forum.

46	0.012856281	0.007572242	0.004947634	0.003282873	0.002157292	0.001418412	0.001289182	0.001289182	0.001289182	0.001289182
47	0.01393607	0.008208229	0.005363182	0.003558599	0.002338481	0.001537544	0.00139746	0.00139746	0.00139746	0.00139746
48	0.015219689	0.008964269	0.005857172	0.003886373	0.002553873	0.001679163	0.001526176	0.001526176	0.001526176	0.001526176
49	0.016706545	0.009840015	0.006429377	0.004266044	0.002803369	0.001843206	0.001675273	0.001675273	0.001675273	0.001675273
50	0.018289462	0.01077234	0.007038549	0.004670245	0.003068983	0.002017846	0.001834002	0.001834002	0.001834002	0.001834002
51	0.01997119	0.011762864	0.007685748	0.005099677	0.003351178	0.002203388	0.00200264	0.00200264	0.00200264	0.00200264
52	0.02186168	0.012876347	0.008413288	0.005582417	0.003668403	0.002411963	0.002192212	0.002192212	0.002192212	0.002192212
53	0.023951075	0.014106983	0.009217374	0.006115947	0.004019005	0.002642483	0.002401729	0.002401729	0.002401729	0.002401729
54	0.026160464	0.015408294	0.010067639	0.006680118	0.004389742	0.002886241	0.002623278	0.002623278	0.002623278	0.002623278
55	0.023667955	0.013940227	0.009108418	0.006043652	0.003971497	0.002611246	0.002373338	0.002373338	0.002373338	0.002373338
56	0.025587569	0.015070864	0.009847165	0.006533828	0.00429361	0.002823034	0.00256583	0.00256583	0.00256583	0.00256583
57	0.02751011	0.016203224	0.010587039	0.007024753	0.004616213	0.003035145	0.002758616	0.002758616	0.002758616	0.002758616
58	0.029461126	0.017352356	0.011337872	0.007522948	0.004943595	0.003250397	0.002954257	0.002954257	0.002954257	0.002954257
59	0.031508268	0.018558106	0.012125698	0.008045689	0.005287107	0.003476255	0.003159537	0.003159537	0.003159537	0.003159537
60	0.033720276	0.01986096	0.012976971	0.008610529	0.005658283	0.003720302	0.003381349	0.003381349	0.003381349	0.003381349
61	0.036125426	0.021277574	0.013902573	0.009224688	0.006061869	0.003985659	0.003622529	0.003622529	0.003622529	0.003622529
62	0.038733632	0.022813785	0.01490632	0.009890698	0.006499527	0.004273418	0.003884071	0.003884071	0.003884071	0.003884071
63	0.041564087	0.024480899	0.015995597	0.010613459	0.006974479	0.004585697	0.004167899	0.004167899	0.004167899	0.004167899
64	0.044651353	0.026299273	0.017183706	0.011401798	0.007492524	0.00492631	0.004477479	0.004477479	0.004477479	0.004477479

© 2022 Avanceña ALV et al. JAMA Health Forum.

Estimation of mortality rates under various scenarios

We estimated two additional sets of age-, gender-, and household income group-specific mortality rates using both the NLMS and PSID studies, which we refer to as the high- and low-effect scenarios. These scenarios reflect the uncertainty in the estimated relationship between household income and mortality in the NLMS and PSID studies. In the high-effect scenario, we assumed that household income has a bigger effect on mortality (i.e., larger difference in mortality between household income groups), while in the low-effect scenario we assumed that household income has a smaller effect on mortality (i.e., smaller difference in mortality between household income groups). Under the high-effect scenario, individuals with lower household income would have greater mortality than in the base case. In contrast, under the low-effect scenario, individuals with lower household income would have smaller mortality than in the base case. We used the mortality rate estimates under the high- and low-effect scenarios in sensitivity analyses to generate different estimates of the number of deaths averted from each policy we modeled.

Using the NLMS, we calculated the age-, gender-, and household income group-specific mortality rates for the high- and low-effect scenarios by applying the 95% confidence intervals reported for the mortality incident rate ratios (r_i) for each household income group to Eq. 1. If household income had a greater effect on mortality (as in the high-effect scenario), we would expect the rate ratios to be further from the reference income group ($26 837-32 466 1990 US$ for males) as the absolute magnitude of the income effect would be greater, and vice versa for the low-effect scenario. For the high-effect scenario, the mortality incident rate ratios (r_i) used in Eq. 1 for the lowest six household income groups were set equal to the higher incident mortality rate ratios reported in the 95% confidence intervals, and the mortality incident rate ratios (r_i) for the top three household income groups were set equal to the lower incident mortality rate ratios reported in the 95% confidence intervals, signifying the greater impact household income may have on mortality. On the other hand, for the low-effect scenario, the mortality incident rate ratios (r_i) used in Eq. 1 for the lowest six household income groups were set equal to the lower incident mortality rate ratios reported in the 95% confidence intervals, and the mortality incident rate ratios (r_i) for the top three household income groups were set equal to the higher incident mortality rate ratios reported in the 95% confidence intervals, signifying the smaller impact household income may have on mortality. The mortality rates under high- and low-effect scenarios using the NLMS are show in eTables 11 and 12.

Using the PSID, we calculated the age-, gender-, and household income group-specific mortality rates for the high- and low-effect scenarios by applying the 95% confidence intervals reported for the mortality hazard rate ratios (Δ) in Eq. 2. For the high-effect scenario, Δ was set at 0.615; for the low-effect scenario, Δ was set at 0.468. Similar to the NLMS study, under the high-effect scenario, the resulting age-, gender-, and household income group-specific mortality rates must be magnified in lower income individuals than the base case, but the adjusted mortality rates must be reduced in higher income individuals, however this change is less apparent as it is logarithmic in the PSID study. The mortality rates under high- and low-effect scenarios using the PSID are show in eTables 13 and 14

eTable 11. Mortality rates estimated using data from the National Longitudinal Mortality Survey under high-effect scenario[8]

Age	Household income (2019 $)									
	1 (≤$11 676)	2 ($11 677-19 915)	3 ($19 916-27 708)	4 ($27 709-35 348)	5 ($35 349-43 505)	6 ($43 506-52 493)	7 ($52 494-63 466)	8 ($63 467-78 567)	9 ($78 568-105 574)	10 (≥$105 575)
					Males					
18	0.0022214	0.001508358	0.00128896	0.001151837	0.001060421	0.000978147	0.000914156	0.000850165	0.000777033	0.000594202
19	0.002588616	0.001757702	0.001502036	0.001342245	0.001235718	0.001139843	0.001065274	0.000990705	0.000905483	0.000692428
20	0.002966176	0.00201407	0.001721115	0.001538017	0.001415952	0.001306094	0.001220649	0.001135203	0.001037551	0.000793422
21	0.003325634	0.002258147	0.001929689	0.001724403	0.001587546	0.001464374	0.001368574	0.001272774	0.001163288	0.000889573
22	0.003573685	0.002426577	0.00207362	0.001853022	0.001705957	0.001573598	0.001470652	0.001367707	0.001250055	0.000955924
23	0.00379529	0.002577048	0.002202205	0.001967928	0.001811743	0.001671177	0.001561848	0.001452518	0.00132757	0.001015201
24	0.003958309	0.00268774	0.002296796	0.002052456	0.001889563	0.001742959	0.001628934	0.001514908	0.001384594	0.001058807
25	0.004184304	0.002841194	0.002427929	0.002169639	0.001997445	0.001842471	0.001721936	0.0016014	0.001463645	0.001119258
26	0.004327335	0.002938314	0.002510923	0.002243803	0.002065724	0.001905452	0.001780796	0.00165614	0.001513677	0.001157518

© 2022 Avanceña ALV et al. JAMA Health Forum.

27	0.004454762	0.003024839	0.002584862	0.002309877	0.002126553	0.001961562	0.001833236	0.001704909	0.00155825	0.001191603
28	0.00458219	0.003111363	0.002658802	0.00237595	0.002187383	0.002017672	0.001885675	0.001753678	0.001602824	0.001225689
29	0.004707017	0.003196123	0.002731232	0.002440675	0.002246971	0.002072637	0.001937044	0.001801451	0.001646487	0.001259079
30	0.004925641	0.003344571	0.002858088	0.002554036	0.002351335	0.002168904	0.002027013	0.001885122	0.001722961	0.001317558
31	0.005052823	0.003430929	0.002931885	0.002619982	0.002412047	0.002224906	0.002079351	0.001933796	0.001767448	0.001351578
32	0.005190603	0.003524484	0.003011831	0.002691424	0.002477819	0.002285574	0.002136051	0.001986527	0.001815643	0.001388433
33	0.005336332	0.003623435	0.00309639	0.002766987	0.002547385	0.002349743	0.002196021	0.0020423	0.001866618	0.001427414
34	0.00549001	0.003727785	0.003185561	0.002846672	0.002620745	0.002417412	0.002259263	0.002101115	0.001920374	0.001468521
35	0.005798869	0.003937504	0.003364776	0.003006821	0.002768185	0.002553412	0.002386366	0.00221932	0.002028411	0.001551138
36	0.005994063	0.004070043	0.003478036	0.003108033	0.002861363	0.002639361	0.002466693	0.002294024	0.002096689	0.00160335
37	0.00617299	0.004191536	0.003581858	0.00320081	0.002946777	0.002718148	0.002540325	0.002362502	0.002159276	0.001651211
38	0.006324807	0.004294622	0.00366995	0.00327953	0.003019249	0.002784997	0.002602801	0.002420605	0.002212381	0.001691821
39	0.006479335	0.004399549	0.003759614	0.003359655	0.003093016	0.002853041	0.002666393	0.002479746	0.002266434	0.001733155
40	0.00667724	0.004533928	0.003874448	0.003462272	0.003187489	0.002940184	0.002747835	0.002555487	0.00233566	0.001786093
41	0.006956474	0.004723532	0.004036473	0.003607061	0.003320786	0.003063139	0.002862747	0.002662354	0.002433335	0.001860785
42	0.007322462	0.004972042	0.004248836	0.003796832	0.003495496	0.003224294	0.003013359	0.002802424	0.002561355	0.001958683
43	0.007780624	0.00528314	0.004514683	0.004034398	0.003714207	0.003426036	0.003201903	0.00297777	0.002721618	0.002081237
44	0.008311984	0.00564394	0.004823003	0.004309918	0.003967861	0.003660009	0.00342057	0.00318113	0.002907484	0.00222337
45	0.008987462	0.006102598	0.005214947	0.004660166	0.004290311	0.003957442	0.003698544	0.003439646	0.003143763	0.002404054
46	0.009645082	0.006549129	0.005596529	0.005001153	0.004604236	0.004247011	0.003969169	0.003691328	0.003373794	0.00257996
47	0.010423264	0.007077525	0.006048067	0.005404656	0.004975715	0.004589668	0.004289409	0.003989151	0.003645998	0.002788116
48	0.011360372	0.007713833	0.006591821	0.005890563	0.005423058	0.005002304	0.00467505	0.004347797	0.003973793	0.003038783
49	0.012459144	0.008459912	0.00722938	0.006460297	0.005947575	0.005486125	0.00512722	0.004768314	0.004358137	0.003332693
50	0.013640118	0.009261809	0.007914636	0.007072654	0.006511332	0.006006143	0.005613217	0.005220292	0.004771235	0.003648591
51	0.014908775	0.010123242	0.008650771	0.007730476	0.007116946	0.006564769	0.006135298	0.005705828	0.005215004	0.003987944
52	0.016344577	0.01109817	0.00948389	0.008474966	0.00780235	0.007196995	0.006726163	0.006255332	0.005717239	0.004372006
53	0.017944784	0.01218473	0.010412405	0.009304703	0.008566234	0.007901613	0.007384685	0.006867757	0.006276982	0.004800045
54	0.019646374	0.01334013	0.011399748	0.010187008	0.009378516	0.008650872	0.008084927	0.007518982	0.006872188	0.005255203
55	0.019891434	0.01350653	0.011541943	0.010314077	0.0094955	0.00875878	0.008185776	0.007612771	0.006957909	0.005320754
56	0.021528023	0.014617793	0.012491569	0.011162679	0.010276752	0.009479418	0.008859269	0.00823912	0.007530378	0.005758525
57	0.023238539	0.015779255	0.01348409	0.012049613	0.011093294	0.010232608	0.009563185	0.008893762	0.008128707	0.00621607
58	0.025079064	0.017028994	0.014552049	0.013003959	0.011971899	0.011043044	0.010320602	0.00959816	0.008772512	0.006708391

© 2022 Avanceña ALV et al. JAMA Health Forum.

59	0.027069991	0.018380858	0.015707279	0.014036292	0.0129223	0.011919708	0.011139914	0.01036012	0.009468927	0.007240944
60	0.029239364	0.019853889	0.01696605	0.015161151	0.013957885	0.012874946	0.01203266	0.011190374	0.010227761	0.007821229
61	0.031510704	0.021396157	0.018283989	0.016338883	0.015042147	0.013875084	0.012967368	0.012059652	0.011022263	0.008428789
62	0.033802438	0.022952273	0.01961376	0.01752719	0.016136143	0.014884201	0.013910468	0.012936735	0.011823898	0.009041804
63	0.036045737	0.0244755	0.020915427	0.018690382	0.017207018	0.015871991	0.014833637	0.013795282	0.012608591	0.009641864
64	0.038286487	0.025996997	0.022215616	0.019852252	0.018276677	0.016858659	0.015755756	0.014652853	0.013392392	0.010241241
					Females					
18	0.000852746	0.000576754	0.000495371	0.000445834	0.000410451	0.000378605	0.000353837	0.000329068	0.000300761	0.000233532
19	0.000961878	0.000650565	0.000558767	0.00050289	0.000462978	0.000427058	0.000399119	0.000371181	0.000339251	0.000263419
20	0.001076085	0.000727808	0.000625111	0.0005626	0.00051795	0.000477764	0.000446508	0.000415253	0.000379532	0.000294695
21	0.001195368	0.000808485	0.000694405	0.000624964	0.000575364	0.000530723	0.000496003	0.000461283	0.000421603	0.000327362
22	0.00123789	0.000837245	0.000719106	0.000647196	0.000595831	0.000549603	0.000513647	0.000477692	0.0004366	0.000339007
23	0.001327173	0.000897631	0.000770972	0.000693874	0.000638805	0.000589243	0.000550694	0.000512145	0.00046809	0.000363458
24	0.001406803	0.000951489	0.00081723	0.000735507	0.000677133	0.000624597	0.000583736	0.000542874	0.000496175	0.000385266
25	0.001492896	0.001009718	0.000867242	0.000780518	0.000718572	0.000662821	0.000619459	0.000576097	0.00052654	0.000408843
26	0.001573264	0.001064075	0.000913929	0.000822536	0.000757256	0.000698503	0.000652806	0.00060711	0.000554886	0.000430852
27	0.001660938	0.001123373	0.00096486	0.000868374	0.000799456	0.000737429	0.000689186	0.000640943	0.000585808	0.000454863
28	0.001763224	0.001192554	0.00102428	0.000921852	0.000848689	0.000782842	0.000731628	0.000680414	0.000621884	0.000482875
29	0.001884994	0.001274913	0.001095017	0.000985515	0.0009073	0.000836906	0.000782155	0.000727404	0.000664832	0.000516222
30	0.002096884	0.001418225	0.001218107	0.001096296	0.001009289	0.000930982	0.000870077	0.000809171	0.000739565	0.000574251
31	0.002241235	0.001515856	0.001301962	0.001171766	0.001078769	0.000995071	0.000929973	0.000864875	0.000790477	0.000613782
32	0.002380521	0.001610062	0.001382875	0.001244588	0.001145811	0.001056912	0.000987768	0.000918624	0.000839603	0.000651927
33	0.002504612	0.001693991	0.001454961	0.001309465	0.001205539	0.001112006	0.001039258	0.00096651	0.000883369	0.00068591
34	0.002623638	0.001774494	0.001524105	0.001371695	0.00126283	0.001164852	0.001088646	0.001012441	0.00092535	0.000718507
35	0.002841353	0.001921745	0.001650578	0.001485521	0.001367622	0.001261514	0.001178985	0.001096456	0.001002137	0.00077813
36	0.002990347	0.002022517	0.001737131	0.001563418	0.001439337	0.001327665	0.001240808	0.001153952	0.001054687	0.000818933
37	0.00314457	0.002126825	0.001826721	0.001644049	0.001513569	0.001396137	0.001304801	0.001213465	0.001109081	0.000861169
38	0.00330402	0.002234669	0.001919348	0.001727413	0.001590317	0.00146693	0.001370963	0.001274995	0.001165318	0.000904835
39	0.003481768	0.002354889	0.002022604	0.001820344	0.001675872	0.001545847	0.001444717	0.001343587	0.00122801	0.000953513
40	0.003696111	0.002499859	0.002147119	0.001932407	0.001779041	0.001641012	0.001533656	0.0014263	0.001303608	0.001012213
41	0.003954891	0.002674885	0.002297447	0.002067703	0.001903599	0.001755906	0.001641034	0.001526161	0.001394879	0.001083082
42	0.004250267	0.002874662	0.002469035	0.002222131	0.002045771	0.001887048	0.001763596	0.001640144	0.001499057	0.001163973

© 2022 Avanceña ALV et al. JAMA Health Forum.

43	0.004574395	0.003093886	0.002657325	0.002391592	0.002201784	0.002030956	0.001898089	0.001765223	0.001613376	0.001252739
44	0.004922049	0.003329021	0.002859282	0.002573354	0.002369119	0.002185308	0.002042344	0.00189938	0.001735992	0.001347947
45	0.005299356	0.003584212	0.003078464	0.002770618	0.002550728	0.002352826	0.002198903	0.00204498	0.001869068	0.001451276
46	0.005712837	0.003863869	0.003318661	0.002986794	0.002749747	0.002536405	0.002370472	0.002204539	0.002014901	0.001564511
47	0.006191742	0.004187776	0.003596863	0.003237176	0.002980258	0.002749031	0.002569188	0.002389344	0.002183809	0.001695664
48	0.006762241	0.004573632	0.003928272	0.003535445	0.003254854	0.003002323	0.002805909	0.002609495	0.002385023	0.0018519
49	0.007421716	0.005019667	0.00431137	0.003880233	0.003572278	0.003295119	0.00307955	0.002863982	0.002617618	0.002032503
50	0.00812568	0.005495792	0.004720312	0.004248281	0.003911116	0.003607667	0.003371651	0.003135636	0.002865904	0.00222529
51	0.008874132	0.006002006	0.005155098	0.004639588	0.004271367	0.003939967	0.003682213	0.003424458	0.003129881	0.00243026
52	0.009711561	0.0065684	0.005641571	0.005077414	0.004674444	0.004311772	0.004029694	0.003747615	0.003425239	0.002659598
53	0.010640584	0.007196744	0.006181252	0.005563127	0.005121609	0.004724243	0.00441518	0.004106118	0.003752903	0.002914019
54	0.011621947	0.007860487	0.006751338	0.006076204	0.005593966	0.005159951	0.004822384	0.004484818	0.004099027	0.003182774
55	0.011578125	0.007830848	0.006725882	0.006053294	0.005572874	0.005140495	0.004804201	0.004467907	0.004083571	0.003170773
56	0.012517994	0.008466527	0.007271864	0.006544677	0.006025258	0.005557781	0.005194188	0.004830595	0.00441506	0.003428164
57	0.013457862	0.009102206	0.007817845	0.007036061	0.006477643	0.005975068	0.005584175	0.005193283	0.004746549	0.003685556
58	0.014412117	0.009747614	0.008372184	0.007534966	0.006936952	0.006398741	0.005980131	0.005561522	0.005083112	0.003946887
59	0.015414323	0.010425455	0.008954379	0.008058941	0.007419342	0.006843704	0.006395985	0.005948266	0.005436587	0.00422135
60	0.016495652	0.01115681	0.009582536	0.008624283	0.007939816	0.007323796	0.006844669	0.006365542	0.005817968	0.004517481
61	0.017672885	0.01195303	0.010266406	0.009239766	0.008506451	0.007846468	0.007333147	0.006819827	0.006233175	0.004839877
62	0.018948421	0.012815737	0.011007381	0.009906643	0.009120402	0.008412784	0.007862415	0.007312046	0.006683053	0.005189194
63	0.020334247	0.013753039	0.011812426	0.010631183	0.009787439	0.009028068	0.008437447	0.007846826	0.00717183	0.005568715
64	0.02184475	0.014774665	0.012689896	0.011420907	0.010514486	0.009698707	0.009064212	0.008429717	0.00770458	0.00598238

© 2022 Avanceña ALV et al. JAMA Health Forum.

eTable 12. Mortality rates estimated using data from the National Longitudinal Mortality Survey under low-effect scenario[8]

Age	Household income (2019 $)									
	1 (≤$11 676)	2 ($11 677-19 915)	3 ($19 916-27 708)	4 ($27 709-35 348)	5 ($35 349-43 505)	6 ($43 506-52 493)	7 ($52 494-63 466)	8 ($63 467-78 567)	9 ($78 568-105 574)	10 (≥$105 575)
Males										
18	0.001861841	0.001369001	0.001204721	0.001104327	0.001031314	0.000967427	0.000912667	0.000857907	0.00079402	0.000647994
19	0.002169619	0.001595308	0.001403871	0.001286882	0.001201799	0.001127351	0.001063539	0.000999726	0.000925279	0.000755113
20	0.002486067	0.00182799	0.001608632	0.001474579	0.001377086	0.00129178	0.00121866	0.001145541	0.001060234	0.000865249
21	0.002787343	0.002049517	0.001803575	0.001653277	0.001543969	0.001448325	0.001366345	0.001284364	0.00118872	0.000970105
22	0.003003415	0.002208393	0.001943386	0.001781437	0.001663656	0.001560598	0.001472262	0.001383926	0.001280868	0.001045306
23	0.003189656	0.002345336	0.002063895	0.001891904	0.001766819	0.00165737	0.001563557	0.001469744	0.001360295	0.001110125
24	0.003326662	0.002446075	0.002152546	0.001973167	0.00184271	0.00172856	0.001630717	0.001532874	0.001418723	0.001157809
25	0.003497974	0.00257204	0.002263395	0.002074779	0.001937603	0.001817575	0.001714693	0.001611812	0.001491783	0.001217432
26	0.003617544	0.002659959	0.002340764	0.0021457	0.002003836	0.001879704	0.001773306	0.001666908	0.001542776	0.001259047
27	0.003724071	0.002738287	0.002409693	0.002208885	0.002062843	0.001935056	0.001825525	0.001715993	0.001588207	0.001296123
28	0.003830597	0.002816615	0.002478621	0.00227207	0.00212185	0.001990408	0.001877744	0.001765079	0.001633637	0.001333198
29	0.003934949	0.002893345	0.002546143	0.002333965	0.002179653	0.00204463	0.001928897	0.001813163	0.00167814	0.001369517
30	0.004100277	0.003014909	0.00265312	0.002432027	0.002271232	0.002130536	0.00200994	0.001889343	0.001748647	0.001427057
31	0.004206147	0.003092755	0.002721625	0.002494823	0.002329876	0.002185547	0.002061837	0.001938127	0.001793798	0.001463904
32	0.00432084	0.003177088	0.002795838	0.002562851	0.002393407	0.002245143	0.002118059	0.001990975	0.001842711	0.001503822
33	0.00444215	0.003266287	0.002874333	0.002634805	0.002460603	0.002308176	0.002177525	0.002046873	0.001894446	0.001546042
34	0.004570077	0.003360351	0.002957109	0.002710683	0.002531464	0.002374648	0.002240234	0.00210582	0.001949003	0.001590566
35	0.004808556	0.003535703	0.003111419	0.002852134	0.002663563	0.002498563	0.002357135	0.002215707	0.002050708	0.001673566
36	0.004970415	0.003654717	0.003216151	0.002948138	0.00275322	0.002582667	0.002436478	0.002290289	0.002119736	0.001729899
37	0.005118786	0.003763813	0.003312155	0.003036142	0.002835406	0.002659761	0.002509209	0.002358656	0.002183011	0.001781538
38	0.005244676	0.003856379	0.003393614	0.003110813	0.002905139	0.002725175	0.002570919	0.002416664	0.0022367	0.001825353
39	0.005372814	0.003950599	0.003476527	0.003186816	0.002976118	0.002791756	0.002633732	0.002475708	0.002291347	0.00186995
40	0.005536921	0.004071265	0.003582713	0.003284154	0.00306702	0.002877027	0.002714177	0.002551326	0.002361334	0.001927066
41	0.005768469	0.004241521	0.003732539	0.003421494	0.003195279	0.002997342	0.002827681	0.00265802	0.002460082	0.002007653
42	0.006071954	0.004464672	0.003928912	0.003601502	0.003363386	0.003155035	0.002976448	0.002797861	0.00258951	0.002113278
43	0.006451873	0.004744024	0.004174741	0.003826846	0.003573832	0.003352444	0.003162683	0.002972922	0.002751534	0.002245505
44	0.006892489	0.005068006	0.004459846	0.004088192	0.003817898	0.003581391	0.003378671	0.003175951	0.002939444	0.002398856

© 2022 Avanceña ALV et al. JAMA Health Forum.

45	0.007440184	0.005470724	0.004814237	0.00441305	0.004121279	0.003865978	0.003647149	0.00342832	0.00317302	0.002589476
46	0.007984588	0.005871021	0.005166498	0.004735957	0.004422836	0.004148855	0.003914014	0.003679173	0.003405192	0.00277895
47	0.008628799	0.006344705	0.005583341	0.005118062	0.004779678	0.004483592	0.004229803	0.003976015	0.003679929	0.00300316
48	0.009404574	0.006915128	0.006085313	0.005578203	0.005209397	0.004886691	0.004610085	0.00433348	0.004010774	0.003273161
49	0.010314182	0.007583958	0.006673883	0.006117726	0.005713248	0.00535933	0.005055972	0.004752613	0.004398695	0.00358974
50	0.011291841	0.008302824	0.007306485	0.006697611	0.006254794	0.005867329	0.005535216	0.005203103	0.004815638	0.003930003
51	0.012342086	0.009075063	0.007986056	0.007320551	0.006836548	0.006413045	0.006050042	0.00568704	0.005263537	0.00429553
52	0.013530701	0.009949045	0.008755159	0.008025563	0.007494947	0.007030658	0.006632697	0.006234735	0.005770446	0.004709215
53	0.014855417	0.0109231	0.009612328	0.008811301	0.008228736	0.007718991	0.007282067	0.006845143	0.006335398	0.005170268
54	0.016264061	0.011958869	0.010523804	0.009646821	0.009009014	0.008450934	0.007972579	0.007494224	0.006936144	0.005660531
55	0.016732905	0.012303607	0.010827174	0.00992491	0.009268717	0.008694549	0.008202405	0.00771026	0.007136092	0.005823707
56	0.018109623	0.013315899	0.011717991	0.010741492	0.010031311	0.009409902	0.008877266	0.00834463	0.007723221	0.006302859
57	0.019548528	0.014373918	0.012649048	0.01159496	0.010828351	0.010157569	0.009582612	0.009007655	0.008336872	0.006803654
58	0.021096799	0.015512352	0.01365087	0.012513297	0.011685972	0.010962062	0.010341568	0.009721074	0.008997164	0.007342513
59	0.022771591	0.016743817	0.014734559	0.013506679	0.012613675	0.011832297	0.011162544	0.010492792	0.009711414	0.007925407
60	0.024596492	0.018085656	0.015915377	0.014589096	0.013624527	0.01278053	0.012057104	0.011333678	0.01048968	0.008560544
61	0.026507169	0.019490566	0.017151698	0.01572239	0.014682893	0.013773333	0.012993711	0.012214088	0.011304528	0.009225534
62	0.028435003	0.02090809	0.018399119	0.016865859	0.015750761	0.01477505	0.013938727	0.013102403	0.012126692	0.009896496
63	0.030322092	0.022295656	0.019620177	0.017985162	0.016796061	0.015755597	0.01486377	0.013971944	0.01293148	0.010553277
64	0.032207037	0.023681644	0.020839847	0.019103193	0.017840172	0.016735029	0.015787763	0.014840497	0.013735354	0.011209312
Females										
18	0.00072503	0.000529557	0.000465583	0.000426489	0.000398056	0.000376732	0.000355407	0.000334083	0.000309204	0.000252339
19	0.000817817	0.000597327	0.000525167	0.000481069	0.000448998	0.000424944	0.000400891	0.000376837	0.000348775	0.000284632
20	0.000914919	0.00066825	0.000587522	0.000538188	0.000502309	0.000475399	0.00044849	0.000421581	0.000390186	0.000318428
21	0.001016337	0.000742325	0.000652648	0.000597845	0.000557989	0.000528097	0.000498205	0.000468312	0.000433438	0.000353725
22	0.001062701	0.000776188	0.000682421	0.000625118	0.000583444	0.000552188	0.000520932	0.000489676	0.000453211	0.000369862
23	0.001139348	0.000832171	0.00073164	0.000670205	0.000625524	0.000592014	0.000558504	0.000524994	0.000485898	0.000396538
24	0.001207709	0.000882101	0.000775539	0.000710417	0.000663056	0.000627535	0.000592014	0.000556493	0.000515052	0.00042033
25	0.001278995	0.000934168	0.000821315	0.00075235	0.000702193	0.000664576	0.000626958	0.000589341	0.000545454	0.00044514
26	0.001347848	0.000984457	0.00086553	0.000792852	0.000739995	0.000700352	0.00066071	0.000621067	0.000574817	0.000469104
27	0.00142296	0.001039319	0.000913763	0.000837035	0.000781233	0.000739381	0.000697529	0.000655678	0.000606851	0.000495246
28	0.001510591	0.001103324	0.000970036	0.000888583	0.000829344	0.000784915	0.000740486	0.000696057	0.000644223	0.000525745

© 2022 Avanceña ALV et al. JAMA Health Forum.

29	0.001614913	0.00117952	0.001037028	0.000949949	0.000886619	0.000839122	0.000791624	0.000744127	0.000688713	0.000562053
30	0.00178225	0.001301741	0.001144484	0.001048382	0.00097849	0.000926071	0.000873652	0.000821233	0.000760077	0.000620293
31	0.001904941	0.001391354	0.001223271	0.001120553	0.00104585	0.000989822	0.000933795	0.000877767	0.000812401	0.000662994
32	0.002023327	0.001477822	0.001299293	0.001190192	0.001110846	0.001051337	0.000991827	0.000932317	0.000862889	0.000704197
33	0.002128798	0.001554858	0.001367022	0.001252234	0.001168752	0.00110614	0.001043529	0.000980917	0.00090787	0.000740905
34	0.002229965	0.001628749	0.001431987	0.001311744	0.001224294	0.001158707	0.00109312	0.001027533	0.000951014	0.000776115
35	0.002400464	0.00175328	0.001541475	0.001412038	0.001317902	0.0012473	0.001176698	0.001106096	0.001023727	0.000835456
36	0.00252634	0.001845219	0.001622306	0.001486082	0.00138701	0.001312706	0.001238402	0.001164098	0.00107741	0.000879265
37	0.002656632	0.001940383	0.001705974	0.001562724	0.001458543	0.001380407	0.00130227	0.001224134	0.001132975	0.000924612
38	0.00279134	0.002038773	0.001792478	0.001641965	0.001532501	0.001450402	0.001368304	0.001286206	0.001190424	0.000971496
39	0.002941507	0.002148454	0.001888909	0.001730298	0.001614945	0.00152843	0.001441915	0.0013554	0.001254466	0.00102376
40	0.003122591	0.002280716	0.002005193	0.001836818	0.001714364	0.001622523	0.001530682	0.001438841	0.001331693	0.001086784
41	0.003341217	0.002440398	0.002145585	0.001965422	0.001834393	0.001736122	0.001637851	0.00153958	0.001424931	0.001162874
42	0.003590759	0.002622662	0.00230583	0.002112211	0.001971397	0.001865786	0.001760176	0.001654565	0.001531353	0.001249725
43	0.003864593	0.002822668	0.002481675	0.00227329	0.002121737	0.002008073	0.001894408	0.001780744	0.001648135	0.00134503
44	0.004158302	0.003037191	0.002670282	0.00244606	0.002282989	0.002160686	0.002038383	0.00191608	0.001773393	0.001447252
45	0.004480425	0.003272467	0.002877136	0.002635544	0.002459841	0.002328064	0.002196287	0.002064509	0.001910769	0.001559364
46	0.004830009	0.0035278	0.003101623	0.002841181	0.002651769	0.00250971	0.002367651	0.002225592	0.002059857	0.001681032
47	0.005234906	0.003823534	0.003361631	0.003079357	0.002874066	0.002720098	0.00256613	0.002412163	0.002232534	0.001821953
48	0.005717243	0.00417583	0.003671367	0.003363084	0.003138879	0.002970724	0.00280257	0.002634416	0.002438236	0.001989825
49	0.006274807	0.00458307	0.004029411	0.003691063	0.003444992	0.003260439	0.003075886	0.002891333	0.002676021	0.002183879
50	0.006869985	0.005017783	0.004411608	0.004041167	0.003771756	0.003569698	0.00336764	0.003165581	0.002929846	0.002391024
51	0.007502776	0.005479968	0.004817959	0.004413397	0.004119171	0.003898501	0.003677831	0.003457161	0.003199713	0.00261126
52	0.008210793	0.005997099	0.005272617	0.004829878	0.004507886	0.004266393	0.004024899	0.003783405	0.003501662	0.002857678
53	0.00899625	0.006570791	0.005777004	0.005291912	0.004939118	0.004674522	0.004409927	0.004145331	0.003836636	0.003131048
54	0.009825959	0.007176803	0.006309807	0.005779976	0.005394644	0.005105645	0.004816646	0.004527648	0.004190482	0.003419819
55	0.009965141	0.007278461	0.006399184	0.005861848	0.005471058	0.005177966	0.004884873	0.004591781	0.00424984	0.00346826
56	0.010774074	0.007869299	0.006918645	0.00633769	0.005915178	0.005598293	0.005281409	0.004964524	0.004594826	0.0037498
57	0.011583006	0.008460137	0.007438107	0.006813533	0.006359298	0.006018621	0.005677944	0.005337268	0.004939811	0.00403134
58	0.01240432	0.009060018	0.00796552	0.007296659	0.006810215	0.006445382	0.006080549	0.005715716	0.005290078	0.00431719
59	0.013266907	0.009690045	0.008519435	0.007804063	0.007283792	0.006893589	0.006503386	0.006113183	0.005657946	0.004617404
60	0.014197592	0.01036981	0.009117081	0.008351525	0.007794756	0.00737718	0.006959604	0.006542028	0.006054855	0.004941319

© 2022 Avanceña ALV et al. JAMA Health Forum.

61	0.015210821	0.011109864	0.009767733	0.008947542	0.008351039	0.007903662	0.007456285	0.007008908	0.006486968	0.005293962
62	0.016308658	0.011911716	0.010472717	0.009593328	0.008953773	0.008474107	0.00799444	0.007514774	0.006955163	0.005676053
63	0.017501421	0.012782901	0.011238658	0.010294954	0.009608623	0.009093876	0.008579128	0.00806438	0.007463841	0.006091181
64	0.018801491	0.013732462	0.012073507	0.011059701	0.010322387	0.009769402	0.009216417	0.008663432	0.008018283	0.006543656

© 2022 Avanceña ALV et al. JAMA Health Forum.

eTable 13. Mortality rates estimated using data from the Panel Study of Income Dynamics under high-effect scenario[9]

Age	Household income (2019 $)									
	1 (≤$11 676)	2 ($11 677-19 915)	3 ($19 916-27 708)	4 ($27 709-35 348)	5 ($35 349-43 505)	6 ($43 506-52 493)	7 ($52 494-63 466)	8 ($63 467-78 567)	9 ($78 568-105 574)	10 (≥$105 575)
					Males					
18	0.003672439548	0.002408421726	0.001715640228	0.001237218935	0.0008853565158	0.0006335798879	0.0005873732462	0.0005873732462	0.0005873732462	0.0005873732462
19	0.004283611498	0.00280923426	0.002001159205	0.001443118446	0.001032698646	0.0007390210395	0.0006851246312	0.0006851246312	0.0006851246312	0.0006851246312
20	0.004906312447	0.003217607625	0.002292064143	0.001652901996	0.001182820203	0.0008464512076	0.0007847199746	0.0007847199746	0.0007847199746	0.0007847199746
21	0.00550029982	0.003607150346	0.002569555064	0.001853012145	0.001326019454	0.0009489276264	0.0008797228431	0.0008797228431	0.0008797228431	0.0008797228431
22	0.005964268926	0.003911425817	0.002786305824	0.002009320058	0.001437873731	0.001028972918	0.0009539304744	0.0009539304744	0.0009539304744	0.0009539304744
23	0.006334839053	0.004154449326	0.002959423722	0.002134162516	0.001527211261	0.001092904747	0.00101319979	0.00101319979	0.00101319979	0.00101319979
24	0.006606197917	0.00433240912	0.003086193456	0.0022255814	0.00159263081	0.001139720363	0.001056601168	0.001056601168	0.001056601168	0.001056601168
25	0.007268245224	0.00476658621	0.003395479689	0.00244862046	0.001752238038	0.001253938679	0.001162489603	0.001162489603	0.001162489603	0.001162489603
26	0.007514261316	0.004927925966	0.003510410132	0.002531501543	0.001811547918	0.001296382086	0.001201837635	0.001201837635	0.001201837635	0.001201837635
27	0.007738470867	0.005074964779	0.003615153292	0.002607036156	0.001865600649	0.001335063366	0.00123769791	0.00123769791	0.00123769791	0.00123769791
28	0.00795638748	0.005217876623	0.003716956604	0.002680450723	0.00191813627	0.00137265897	0.001272551687	0.001272551687	0.001272551687	0.001272551687
29	0.008176621354	0.005362308148	0.003819842462	0.002754645959	0.00197123054	0.001410654355	0.001307776089	0.001307776089	0.001307776089	0.001307776089
30	0.008660169458	0.005679423718	0.004045739871	0.002917549899	0.002087804947	0.001494077471	0.001385115202	0.001385115202	0.001385115202	0.001385115202
31	0.008887067412	0.005828225613	0.004151738963	0.002993990216	0.002142505802	0.001533222562	0.001421405463	0.001421405463	0.001421405463	0.001421405463
32	0.009128127775	0.005986315354	0.004264354256	0.003075201749	0.002200620949	0.001574810993	0.001459960872	0.001459960872	0.001459960872	0.001459960872
33	0.009384061083	0.006154158916	0.004383917689	0.003161423872	0.002262321684	0.001618965347	0.001500895072	0.001500895072	0.001500895072	0.001500895072
34	0.009651949805	0.006329842957	0.004509066289	0.00325167369	0.002326904647	0.001665182284	0.001543741433	0.001543741433	0.001543741433	0.001543741433
35	0.009960838146	0.006532414948	0.004653368532	0.003355735989	0.002401371851	0.001718472594	0.001593145308	0.001593145308	0.001593145308	0.001593145308
36	0.01029760815	0.006753272008	0.004810696151	0.003469191425	0.002482560803	0.001776573129	0.001647008603	0.001647008603	0.001647008603	0.001647008603
37	0.01060253644	0.006953246956	0.004953148389	0.00357191961	0.002556073312	0.001829180238	0.001695779104	0.001695779104	0.001695779104	0.001695779104
38	0.01086385583	0.007124622768	0.005075228021	0.003659956268	0.002619072533	0.001874263816	0.00173757476	0.00173757476	0.00173757476	0.00173757476
39	0.01112884644	0.007298406195	0.005199022718	0.00374922973	0.002682956813	0.001919980761	0.001779957593	0.001779957593	0.001779957593	0.001779957593
40	0.01146790418	0.00752076357	0.005357419088	0.003863455886	0.002764697295	0.001978475982	0.001834186789	0.001834186789	0.001834186789	0.001834186789
41	0.01194697961	0.007834945922	0.005581227019	0.004024853016	0.002880193426	0.002061127462	0.001910810541	0.001910810541	0.001910810541	0.001910810541
42	0.01257775752	0.008248616236	0.00587590524	0.004237357637	0.003032262187	0.00216995109	0.00201169772	0.00201169772	0.00201169772	0.00201169772
43	0.01336341687	0.00876385931	0.00624293886	0.004502040719	0.003221669967	0.002305495312	0.002137356775	0.002137356775	0.002137356775	0.002137356775

© 2022 Avanceña ALV et al. JAMA Health Forum.

44	0.01427779948	0.009363520354	0.006670107657	0.004810089759	0.003442110519	0.002463247243	0.002283603942	0.002283603942	0.002283603942	0.002283603942
45	0.01520039655	0.009968568528	0.007101113978	0.005120906194	0.003664531425	0.002622416358	0.002431164938	0.002431164938	0.002431164938	0.002431164938
46	0.01631254153	0.01069792407	0.007620670703	0.00549557998	0.003932648787	0.002814286825	0.002609042395	0.002609042395	0.002609042395	0.002609042395
47	0.01762778179	0.01156047148	0.008235106712	0.00593867513	0.004249728624	0.003041195877	0.002819403091	0.002819403091	0.002819403091	0.002819403091
48	0.01921290296	0.01260000942	0.008975622003	0.006472691251	0.004631871702	0.003314665563	0.00307292878	0.00307292878	0.00307292878	0.00307292878
49	0.02107076644	0.01381841444	0.009843553329	0.00709859233	0.005079767853	0.003635189542	0.003370077117	0.003370077117	0.003370077117	0.003370077117
50	0.02306996754	0.01512951005	0.01077751284	0.007772109056	0.005561737863	0.003980097496	0.003689831119	0.003689831119	0.003689831119	0.003689831119
51	0.02521386832	0.01653550112	0.01177907117	0.008494374078	0.006078592257	0.004349969455	0.004032728514	0.004032728514	0.004032728514	0.004032728514
52	0.02764290653	0.0181284881	0.01291383611	0.009312699888	0.006664187957	0.00476903415	0.004421231045	0.004421231045	0.004421231045	0.004421231045
53	0.0303465925	0.01990159177	0.01417690726	0.01022355259	0.007315996097	0.00523548187	0.004853660983	0.004853660983	0.004853660983	0.004853660983
54	0.03322619253	0.02179006159	0.01552215953	0.01119366949	0.008010213828	0.005732278792	0.005314226772	0.005314226772	0.005314226772	0.005314226772
55	0.03201465267	0.02099552192	0.01495616886	0.01078551027	0.007718134219	0.005523260434	0.00512045201	0.00512045201	0.00512045201	0.00512045201
56	0.03464650103	0.02272151378	0.01618567989	0.01167216139	0.008352623653	0.005977314521	0.005541392176	0.005541392176	0.005541392176	0.005541392176
57	0.03740159578	0.02452833183	0.01747276748	0.01260033334	0.00901682549	0.006452631433	0.00598204448	0.00598204448	0.00598204448	0.00598204448
58	0.04036339906	0.02647071135	0.01885642235	0.01359814394	0.009730860887	0.006963610297	0.00645575793	0.00645575793	0.00645575793	0.00645575793
59	0.04356862687	0.02857273105	0.02035379697	0.01467796254	0.01050358139	0.007516585465	0.006968404915	0.006968404915	0.006968404915	0.006968404915
60	0.04705838936	0.03086135138	0.02198409662	0.01585363887	0.01134489788	0.008118649378	0.00752656063	0.00752656063	0.00752656063	0.00752656063
61	0.05071553463	0.03325974297	0.02369259187	0.01708570527	0.01222656723	0.008749590653	0.008111487695	0.008111487695	0.008111487695	0.008111487695
62	0.05440411527	0.03567875018	0.02541577268	0.01832836202	0.01311581506	0.00938595525	0.008701442563	0.008701442563	0.008701442563	0.008701442563
63	0.05801207279	0.03804488397	0.02710128907	0.01954385742	0.01398562617	0.01000841051	0.009278502496	0.009278502496	0.009278502496	0.009278502496
64	0.0616189375	0.04041030107	0.02878629494	0.02075898465	0.01485517382	0.01063067724	0.009855387645	0.009855387645	0.009855387645	0.009855387645
Females										
18	0.00141065665	0.0009251224093	0.0006590113368	0.0004752402577	0.0003400829448	0.0002433705634	0.0002256216786	0.0002256216786	0.0002256216786	0.0002256216786
19	0.001592933871	0.001044661592	0.0007441651236	0.0005366481658	0.0003840265752	0.0002748175567	0.0002547752595	0.0002547752595	0.0002547752595	0.0002547752595
20	0.001783798738	0.001169832635	0.0008333307691	0.0006009491906	0.0004300405261	0.0003077461152	0.0002853023559	0.0002853023559	0.0002853023559	0.0002853023559
21	0.001977643123	0.001296957677	0.0009238883457	0.0006662539944	0.0004767727833	0.000341188709	0.0003163059993	0.0003163059993	0.0003163059993	0.0003163059993
22	0.002029145552	0.001330733473	0.0009479485483	0.0006836047989	0.0004891890562	0.000350074057	0.0003245433436	0.0003245433436	0.0003245433436	0.0003245433436
23	0.002175018414	0.00142639832	0.001016095442	0.0007327483351	0.0005243562758	0.0003752404647	0.0003478743787	0.0003478743787	0.0003478743787	0.0003478743787
24	0.002304316456	0.001511193239	0.001076499139	0.000776307932	0.0005555276165	0.0003975473368	0.0003685544226	0.0003685544226	0.0003685544226	0.0003685544226
25	0.002490212425	0.001633105631	0.001163343482	0.0008389349704	0.0006003436591	0.0004296186468	0.0003982867891	0.0003982867891	0.0003982867891	0.0003982867891
26	0.002621042541	0.001718905299	0.001224462912	0.0008830107122	0.0006318843541	0.0004521898367	0.0004192118742	0.0004192118742	0.0004192118742	0.0004192118742
27	0.002767346537	0.001814852889	0.001292811218	0.0009322994946	0.0006671555123	0.000477430625	0.0004426118654	0.0004426118654	0.0004426118654	0.0004426118654

© 2022 Avanceña ALV et al. JAMA Health Forum.

28	0.002940934501	0.001928693571	0.001373905676	0.0009907800533	0.0007090043251	0.0005073785225	0.0004703756787	0.0004703756787	0.0004703756787	0.0004703756787
29	0.003140945895	0.002059862997	0.001467344271	0.001058162478	0.0007572233327	0.0005418850663	0.0005023656788	0.0005023656788	0.0005023656788	0.0005023656788
30	0.003589732459	0.002354181609	0.001677002258	0.001209355501	0.0008654173829	0.0006193110218	0.0005741450009	0.0005741450009	0.0005741450009	0.0005741450009
31	0.003833567667	0.00251409112	0.001790913865	0.001291501859	0.0009242014928	0.0006613781768	0.0006131442208	0.0006131442208	0.0006131442208	0.0006131442208
32	0.004070902284	0.00266973748	0.001901788615	0.001371458215	0.0009814184318	0.000702323831	0.0006511037304	0.0006511037304	0.0006511037304	0.0006511037304
33	0.00428638171	0.002811050992	0.002002453355	0.001444051711	0.001033366493	0.0007394989646	0.0006855677014	0.0006855677014	0.0006855677014	0.0006855677014
34	0.004487043502	0.002942646955	0.002096195795	0.001511653251	0.001081742299	0.000774117717	0.0007176617267	0.0007176617267	0.0007176617267	0.0007176617267
35	0.004836648393	0.003171921258	0.00225951944	0.001629432668	0.001166025502	0.0008344325635	0.0007735778438	0.0007735778438	0.0007735778438	0.0007735778438
36	0.005092806221	0.003339912063	0.002379187761	0.001715730431	0.001227780366	0.0008786256525	0.0008145479546	0.0008145479546	0.0008145479546	0.0008145479546
37	0.005354168992	0.003511316321	0.002501287656	0.001803781702	0.001290790043	0.0009237167135	0.0008563505486	0.0008563505486	0.0008563505486	0.0008563505486
38	0.005626363434	0.003689824095	0.002628447744	0.001895482086	0.001356411035	0.0009706764856	0.0008998855697	0.0008998855697	0.0008998855697	0.0008998855697
39	0.005930509855	0.003889286289	0.002770534722	0.001997946866	0.00142973505	0.001023148705	0.0009485310186	0.0009485310186	0.0009485310186	0.0009485310186
40	0.006294783865	0.004128180742	0.002940711286	0.002120668206	0.001517554704	0.001085994311	0.001006793328	0.001006793328	0.001006793328	0.001006793328
41	0.006734366073	0.004416463048	0.003146069307	0.002268760346	0.001623529756	0.001161832304	0.001077100497	0.001077100497	0.001077100497	0.001077100497
42	0.007238751768	0.004747244113	0.003381701338	0.002438684323	0.001745127717	0.001248850382	0.001157772393	0.001157772393	0.001157772393	0.001157772393
43	0.007790704272	0.005109219957	0.003639554982	0.002624633221	0.001878193146	0.001344074824	0.001246052167	0.001246052167	0.001246052167	0.001246052167
44	0.008382740281	0.005497482954	0.003916134291	0.002824085969	0.002020921959	0.001446214588	0.001340742933	0.001340742933	0.001340742933	0.001340742933
45	0.008807206542	0.005775852074	0.004114430651	0.002967085653	0.002123252839	0.001519444734	0.001408632444	0.001408632444	0.001408632444	0.001408632444
46	0.009493764053	0.006226103195	0.004435167226	0.003198382028	0.002288769019	0.001637891621	0.001518441063	0.001518441063	0.001518441063	0.001518441063
47	0.0102911377	0.00674902862	0.004807673372	0.003467011575	0.002481000898	0.001775456827	0.001645973713	0.001645973713	0.001645973713	0.001645973713
48	0.01123903032	0.007370665862	0.005250496923	0.003786349902	0.002709520088	0.001938990003	0.001797580502	0.001797580502	0.001797580502	0.001797580502
49	0.01233700442	0.008090727999	0.005763433488	0.004156249617	0.002974221115	0.002128415669	0.001973191455	0.001973191455	0.001973191455	0.001973191455
50	0.01350591458	0.008857310697	0.006309508995	0.004550047189	0.0032560235	0.002330079428	0.002160147984	0.002160147984	0.002160147984	0.002160147984
51	0.01474779237	0.009671746282	0.006889672527	0.004968427043	0.003555417018	0.002544331775	0.002358775022	0.002358775022	0.002358775022	0.002358775022
52	0.01614383099	0.01058728205	0.007541854812	0.00543874259	0.003891975829	0.002785180394	0.00258205868	0.00258205868	0.00258205868	0.00258205868
53	0.0176867518	0.01159914459	0.008262655518	0.00595854171	0.004263945191	0.003051369554	0.002828834807	0.002828834807	0.002828834807	0.002828834807
54	0.01931828218	0.01266911814	0.009024851631	0.006508192766	0.004657276662	0.003332845891	0.003089783226	0.003089783226	0.003089783226	0.003089783226
55	0.01831554605	0.01201151399	0.008556407037	0.006170378047	0.004415535727	0.003159850954	0.002929404717	0.002929404717	0.002929404717	0.002929404717
56	0.01980104687	0.01298571994	0.009250383055	0.006670832778	0.004773662203	0.003416133851	0.00316699704	0.00316699704	0.00316699704	0.00316699704
57	0.02128881354	0.01396141186	0.0099454176	0.007172050857	0.005132334932	0.003672807658	0.003404951764	0.003404951764	0.003404951764	0.003404951764
58	0.02279861527	0.01495155458	0.01065074618	0.007680692393	0.005496319903	0.003933283018	0.003646430795	0.003646430795	0.003646430795	0.003646430795
59	0.02438280498	0.01599048167	0.01139082633	0.008214394713	0.005878238425	0.004206592007	0.003899807506	0.003899807506	0.003899807506	0.003899807506

© 2022 Avanceña ALV et al. JAMA Health Forum.

60	0.02609457624	0.01711307797	0.01219050828	0.008791078354	0.006290914471	0.004501911732	0.004173589722	0.004173589722	0.004173589722	0.004173589722
61	0.02795581221	0.01833369471	0.01306001512	0.009418115603	0.006739623668	0.004823017544	0.004471277459	0.004471277459	0.004471277459	0.004471277459
62	0.02997418289	0.01965736191	0.01400293001	0.01009809042	0.007226215102	0.005171232687	0.004794097458	0.004794097458	0.004794097458	0.004794097458
63	0.03216454254	0.02109382116	0.01502619236	0.01083600711	0.007754269863	0.005549119867	0.005144425529	0.005144425529	0.005144425529	0.005144425529
64	0.0345536363	0.02266061219	0.01614229661	0.01164087592	0.008330235703	0.005961293229	0.005526539309	0.005526539309	0.005526539309	0.005526539309

© 2022 Avanceña ALV et al. JAMA Health Forum.

eTable 14. Mortality rates estimated using data from the Panel Study of Income Dynamics under low-effect scenario[9]

Age	Household income (2019 $)									
	1 (≤$11 676)	2 ($11 677-19 915)	3 ($19 916-27 708)	4 ($27 709-35 348)	5 ($35 349-43 505)	6 ($43 506-52 493)	7 ($52 494-63 466)	8 ($63 467-78 567)	9 ($78 568-105 574)	10 (≥$105 575)
Males										
18	0.00645295061	0.003409163613	0.002041067941	0.001244867884	0.000750454527	0.0004529863289	0.0004034627048	0.0004034627048	0.0004034627048	0.0004034627048
19	0.007526858663	0.003976520854	0.002380745001	0.001452040343	0.0008753461012	0.0005283728762	0.000470607469	0.000470607469	0.000470607469	0.000470607469
20	0.008621024658	0.004554580586	0.002726829648	0.001663120853	0.0010025936	0.0006051814971	0.0005390188359	0.0005390188359	0.0005390188359	0.0005390188359
21	0.00966473719	0.005105985207	0.003056955868	0.001864468158	0.001123973546	0.0006784483694	0.0006042756629	0.0006042756629	0.0006042756629	0.0006042756629
22	0.01044133012	0.005516267653	0.003302592171	0.002014284212	0.00121428846	0.0007329638929	0.0006528311692	0.0006528311692	0.0006528311692	0.0006528311692
23	0.01109006764	0.005859002702	0.003507787814	0.0021394351	0.001289734258	0.0007785041806	0.000693392675	0.000693392675	0.000693392675	0.000693392675
24	0.01156512125	0.006109978663	0.003658047247	0.002231079841	0.001344981253	0.0008118521487	0.0007230948107	0.0007230948107	0.0007230948107	0.0007230948107
25	0.01342280539	0.007091413288	0.004245632642	0.002589454088	0.001561023116	0.0009422584647	0.000839244199	0.000839244199	0.000839244199	0.000839244199
26	0.01387714148	0.00733144396	0.004389339124	0.002677102118	0.001613860739	0.0009741520977	0.0008676509976	0.0008676509976	0.0008676509976	0.0008676509976
27	0.01429120582	0.00755019863	0.004520307653	0.002756981142	0.001662014906	0.001003218721	0.0008935398551	0.0008935398551	0.0008935398551	0.0008935398551
28	0.01469364852	0.007762813466	0.00464760026	0.002834618185	0.001708817519	0.001031469525	0.0009187020845	0.0009187020845	0.0009187020845	0.0009187020845
29	0.01510037069	0.007977689185	0.004776246459	0.002913080797	0.001756117818	0.00106002074	0.0009441318815	0.0009441318815	0.0009441318815	0.0009441318815
30	0.01646395071	0.008698083263	0.00520754675	0.003176135186	0.001914697181	0.001155741775	0.001029388025	0.001029388025	0.001029388025	0.001029388025
31	0.0168953091	0.008925974565	0.005343985385	0.003259350483	0.001964862582	0.001186022412	0.001056358172	0.001056358172	0.001056358172	0.001056358172
32	0.01735359181	0.00916809028	0.005488940183	0.003347759874	0.002018159184	0.001218193091	0.001085011728	0.001085011728	0.001085011728	0.001085011728
33	0.01784014965	0.009425144051	0.005642838403	0.003441623947	0.002074744079	0.001252348637	0.001115433151	0.001115433151	0.001115433151	0.001115433151
34	0.01834943608	0.009694205577	0.005803925671	0.003539872694	0.002133972225	0.001288099691	0.001147275651	0.001147275651	0.001147275651	0.001147275651
35	0.0186956895	0.009877135007	0.005913445612	0.003606670008	0.002174240231	0.001312406102	0.001168924715	0.001168924715	0.001168924715	0.001168924715
36	0.01932777963	0.01021107506	0.006113375687	0.003728609374	0.002247749998	0.00135677777	0.001208445364	0.001208445364	0.001208445364	0.001208445364
37	0.01990010542	0.01051344097	0.006294402304	0.00383901933	0.002314309392	0.001396954083	0.001244229321	0.001244229321	0.001244229321	0.001244229321
38	0.02039058084	0.01077256444	0.006449539654	0.003933639161	0.002371349887	0.001431384636	0.001274895686	0.001274895686	0.001274895686	0.001274895686
39	0.02088794683	0.01103532827	0.006606856489	0.004029588283	0.00242919173	0.001466298895	0.001305992876	0.001305992876	0.001305992876	0.001305992876
40	0.02152433084	0.01137153683	0.006808144719	0.004152356001	0.00250320086	0.001510971988	0.001345781994	0.001345781994	0.001345781994	0.001345781994
41	0.02242351678	0.01184658649	0.007092557187	0.004325822028	0.002607772893	0.001574093335	0.001402002476	0.001402002476	0.001402002476	0.001402002476
42	0.02360743603	0.01247206383	0.00746703078	0.004554217242	0.002745458366	0.001657202484	0.001476025553	0.001476025553	0.001476025553	0.001476025553
43	0.02508205523	0.01325112111	0.007933452757	0.004838692701	0.002916951179	0.001760718283	0.001568224283	0.001568224283	0.001568224283	0.001568224283
44	0.0267982776	0.01415781995	0.008476293805	0.005169776921	0.003116541557	0.00188119422	0.001675528951	0.001675528951	0.001675528951	0.001675528951

© 2022 Avanceña ALV et al. JAMA Health Forum.

45	0.02832817015	0.01496607874	0.008960198738	0.005464915411	0.003294462459	0.001988590116	0.001771183578	0.001771183578	0.001771183578	0.001771183578
46	0.03040081557	0.01606107973	0.009615776374	0.005864758811	0.003535503531	0.002134086354	0.001900773153	0.001900773153	0.001900773153	0.001900773153
47	0.03285195885	0.01735604524	0.01039107287	0.006337619946	0.003820562519	0.002306152508	0.002054027835	0.002054027835	0.002054027835	0.002054027835
48	0.03580606481	0.01891673138	0.01132545643	0.006907509887	0.004164114226	0.002513525801	0.002238729634	0.002238729634	0.002238729634	0.002238729634
49	0.03926846612	0.02074595544	0.01242061378	0.00757545738	0.004566778819	0.002756580576	0.002455211965	0.002455211965	0.002455211965	0.002455211965
50	0.04299427082	0.02271433837	0.01359908561	0.008294219215	0.005000076263	0.003018125828	0.002688163267	0.002688163267	0.002688163267	0.002688163267
51	0.0469897446	0.02482519039	0.01486285376	0.009065004131	0.005464735234	0.003298601398	0.002937975292	0.002937975292	0.002937975292	0.002937975292
52	0.05151661385	0.02721678439	0.01629470227	0.009938302949	0.005991193551	0.00361638004	0.003221012157	0.003221012157	0.003221012157	0.003221012157
53	0.05655532952	0.02987879238	0.01788844777	0.01091034438	0.006577177733	0.003970089444	0.003536051583	0.003536051583	0.003536051583	0.003536051583
54	0.06192188686	0.03271400268	0.01958588958	0.01194563122	0.0072012887	0.004346812784	0.0038715889	0.0038715889	0.0038715889	0.0038715889
55	0.05450248164	0.02879425064	0.01723913209	0.01051432021	0.006338439041	0.003825982959	0.003407699824	0.003407699824	0.003407699824	0.003407699824
56	0.05898300086	0.03116135741	0.01865632009	0.01137867743	0.006859507021	0.004140507907	0.003687838711	0.003687838711	0.003687838711	0.003687838711
57	0.06367333759	0.03363931304	0.02013987335	0.0122835115	0.007404976007	0.004469761693	0.003981096177	0.003981096177	0.003981096177	0.003981096177
58	0.06871557967	0.03630318408	0.02173473426	0.01325623322	0.007991370298	0.004823718645	0.004296356087	0.004296356087	0.004296356087	0.004296356087
59	0.07417223327	0.0391859932	0.0234606735	0.01430890094	0.00862595913	0.005206766591	0.004637526561	0.004637526561	0.004637526561	0.004637526561
60	0.08011328525	0.04232471524	0.02533982794	0.01545501615	0.009316881722	0.005623818494	0.005008983441	0.005008983441	0.005008983441	0.005008983441
61	0.08633929354	0.04561398277	0.02730911403	0.01665610356	0.01004094369	0.006060873852	0.00539825686	0.00539825686	0.00539825686	0.00539825686
62	0.09261881814	0.04893152354	0.02929532733	0.01786751505	0.0107712294	0.006501685965	0.005790876319	0.005790876319	0.005790876319	0.005790876319
63	0.09876108807	0.05217655119	0.03123812699	0.01905244812	0.01148555291	0.00693286303	0.006174914101	0.006174914101	0.006174914101	0.006174914101
64	0.1049014976	0.05542059596	0.0331803382	0.02023702229	0.01219966005	0.007363909498	0.006558835563	0.006558835563	0.006558835563	0.006558835563
Females										
18	0.002453813442	0.001296376186	0.0007761410633	0.0004733762478	0.0002853695181	0.0001722535953	0.0001534216311	0.0001534216311	0.0001534216311	0.0001534216311
19	0.002770881594	0.00146388672	0.0008764297032	0.0005345432988	0.0003222433831	0.0001945112487	0.0001732459227	0.0001732459227	0.0001732459227	0.0001732459227
20	0.003102887808	0.001639289195	0.0009814432515	0.0005985921191	0.0003608544902	0.0002178175291	0.000194004198	0.000194004198	0.000194004198	0.000194004198
21	0.003440076846	0.001817429811	0.001088096127	0.0006636407813	0.0004000683406	0.000241487635	0.0002150865229	0.0002150865229	0.0002150865229	0.0002150865229
22	0.003386480207	0.001789114127	0.001071143512	0.0006533012112	0.0003938352477	0.0002377252407	0.0002117354597	0.0002117354597	0.0002117354597	0.0002117354597
23	0.003629930242	0.001917731414	0.001148146746	0.0007002662585	0.0004221475954	0.0002548150256	0.0002269568701	0.0002269568701	0.0002269568701	0.0002269568701
24	0.003845718243	0.002031734549	0.001216400479	0.0007418948975	0.0004472429497	0.0002699629819	0.0002404487463	0.0002404487463	0.0002404487463	0.0002404487463
25	0.004210393674	0.002224396524	0.001331747299	0.0008122460841	0.000489653367	0.0002955625866	0.0002632496237	0.0002632496237	0.0002632496237	0.0002632496237
26	0.004431598214	0.002341261276	0.001401714283	0.0008549196523	0.000515378645	0.000311090775	0.0002770801622	0.0002770801622	0.0002770801622	0.0002770801622
27	0.004678965632	0.002471948159	0.001479956585	0.0009026404196	0.00054414657	0.0003284555536	0.0002925465021	0.0002925465021	0.0002925465021	0.0002925465021
28	0.004972464155	0.002627006603	0.001572790152	0.0009592605468	0.0005782793734	0.0003490586585	0.0003108971319	0.0003108971319	0.0003108971319	0.0003108971319

© 2022 Avanceña ALV et al. JAMA Health Forum.

29	0.005310638801	0.002805667927	0.001679754775	0.001024499347	0.0006176078464	0.0003727979524	0.0003320410807	0.0003320410807	0.0003320410807	0.0003320410807
30	0.006374576683	0.00336775782	0.002016278272	0.001229748415	0.0007413399263	0.0004474846105	0.0003985624725	0.0003985624725	0.0003985624725	0.0003985624725
31	0.006807574476	0.003596515238	0.002153235451	0.001313279978	0.0007916959841	0.0004778803305	0.0004256351206	0.0004256351206	0.0004256351206	0.0004256351206
32	0.007229028647	0.003819174035	0.002286541383	0.001394584608	0.0008407095609	0.0005074657077	0.000451986018	0.000451986018	0.000451986018	0.000451986018
33	0.007611672796	0.004021329079	0.002407571708	0.001468402221	0.0008852096744	0.0005343266864	0.0004759103672	0.0004759103672	0.0004759103672	0.0004759103672
34	0.007968004081	0.004209582751	0.002520279274	0.001537143701	0.000926649698	0.000559340546	0.0004981895372	0.0004981895372	0.0004981895372	0.0004981895372
35	0.008793128232	0.004645504766	0.002781266	0.001696322132	0.001022608615	0.0006172628799	0.0005497793977	0.0005497793977	0.0005497793977	0.0005497793977
36	0.009258828536	0.004891539274	0.002928566982	0.001786162484	0.001076767855	0.0006499542615	0.0005788967295	0.0005788967295	0.0005788967295	0.0005788967295
37	0.009733991537	0.005142573028	0.003078861014	0.001877828327	0.001132027573	0.0006833099086	0.0006086057047	0.0006086057047	0.0006086057047	0.0006086057047
38	0.01022884674	0.005404010386	0.00323538369	0.001973293082	0.001189577421	0.0007180479155	0.0006395459105	0.0006395459105	0.0006395459105	0.0006395459105
39	0.01078179132	0.005696136986	0.003410280029	0.002079964121	0.00125388285	0.0007568636988	0.0006741180817	0.0006741180817	0.0006741180817	0.0006741180817
40	0.01144404911	0.00604601494	0.003619752134	0.002207723266	0.001330901006	0.0008033530869	0.0007155249257	0.0007155249257	0.0007155249257	0.0007155249257
41	0.01224321878	0.006468224924	0.00387252946	0.002361894702	0.001423841513	0.00085945346	0.0007654920148	0.0007654920148	0.0007654920148	0.0007654920148
42	0.01316020255	0.006952677372	0.00416257138	0.002538794188	0.001530483368	0.0009238241857	0.0008228252839	0.0008228252839	0.0008228252839	0.0008228252839
43	0.01416366378	0.007482816795	0.004479966115	0.002732376432	0.001647182235	0.000994265484	0.0008855654483	0.0008855654483	0.0008855654483	0.0008855654483
44	0.01523999767	0.008051455628	0.004820410465	0.002940016869	0.001772355924	0.00106982232	0.0009528618845	0.0009528618845	0.0009528618845	0.0009528618845
45	0.01559856723	0.008240891808	0.004933826	0.003009190145	0.001814056251	0.001094993304	0.0009752810002	0.0009752810002	0.0009752810002	0.0009752810002
46	0.01681453888	0.008883302785	0.005318437769	0.003243768735	0.001955469302	0.001180352478	0.001051308114	0.001051308114	0.001051308114	0.001051308114
47	0.01822677855	0.009629404278	0.005765129102	0.003516210276	0.002119707604	0.001279489338	0.001139606641	0.001139606641	0.001139606641	0.001139606641
48	0.01990560449	0.01051634619	0.00629614166	0.003840080181	0.002314948914	0.001397340108	0.001244573144	0.001244573144	0.001244573144	0.001244573144
49	0.02185024184	0.01154371913	0.006911230351	0.004215228965	0.002541103116	0.001533850394	0.001366159173	0.001366159173	0.001366159173	0.001366159173
50	0.02392051504	0.01263746686	0.007566057655	0.00461461473	0.002781868308	0.001679180106	0.001495600428	0.001495600428	0.001495600428	0.001495600428
51	0.02612002224	0.01379949031	0.008261761669	0.005038931611	0.003037662942	0.001833581829	0.001633121878	0.001633121878	0.001633121878	0.001633121878
52	0.02859256585	0.01510576184	0.009043827084	0.005515921181	0.003325210711	0.002007150251	0.00178771459	0.00178771459	0.00178771459	0.00178771459
53	0.03132525458	0.01654947086	0.009908176387	0.006043096522	0.003643012403	0.002198980425	0.001958572551	0.001958572551	0.001958572551	0.001958572551
54	0.03421488095	0.01807609173	0.01082216506	0.006600547413	0.003979065369	0.002401827358	0.002139242843	0.002139242843	0.002139242843	0.002139242843
55	0.0294992196	0.01558475683	0.009330601618	0.005690827856	0.003430651222	0.002070795827	0.001844401987	0.001844401987	0.001844401987	0.001844401987
56	0.03189178354	0.01684877424	0.01008736947	0.006152388186	0.003708897647	0.002238749809	0.00199399407	0.00199399407	0.00199399407	0.00199399407
57	0.03428799688	0.01811471967	0.01084529162	0.006614652537	0.003987568484	0.002406959974	0.002143814326	0.002143814326	0.002143814326	0.002143814326
58	0.03671970013	0.0193994148	0.01161443923	0.007083763408	0.004270366668	0.002577661471	0.002295853545	0.002295853545	0.002295853545	0.002295853545
59	0.03927121348	0.0207474069	0.01242148277	0.007575987387	0.004567098327	0.002756773436	0.002455383741	0.002455383741	0.002455383741	0.002455383741
60	0.04202821106	0.02220395855	0.01329352096	0.008107852258	0.004887727051	0.00295031005	0.002627761583	0.002627761583	0.002627761583	0.002627761583

© 2022 Avanceña ALV et al. JAMA Health Forum.

61	0.04502593816	0.02378769021	0.01424170189	0.00868615735	0.005236351736	0.003160745474	0.002815190739	0.002815190739	0.002815190739	0.002815190739
62	0.04827674813	0.02550512828	0.01526993291	0.009313285802	0.005614409032	0.003388946891	0.003018443586	0.003018443586	0.003018443586	0.003018443586
63	0.05180456545	0.02736891233	0.01638578134	0.009993852993	0.006024681267	0.00363659376	0.003239015972	0.003239015972	0.003239015972	0.003239015972
64	0.0556524661	0.02940179987	0.01760287212	0.01073616891	0.006472178023	0.003906709943	0.003479601171	0.003479601171	0.003479601171	0.003479601171

© 2022 Avanceña ALV et al. JAMA Health Forum.

D. Modeled policies

We modeled four hypothetical income support policies (s_a) in the US, in addition to a no-intervention scenario (s_0) where we assumed that no policies or interventions to increase household incomes are implemented. (eTable 15). For each policy scenario, we estimated the associated income gain and recalculated the number of people that fall in each household income group ($l_{igx|s_a}$). These new population numbers were then entered into the life-table model to estimate mortality in the population.

eTable 15. Modeled policy scenarios

**Policy (s_a)**	**Description**	**Projected change in income**	**Affected income groups**
Policy 1: UBI (s_1)	Unconditional basic income guarantee where every adult is given a flat-rate transfer regardless of current income	$1 000 per month ($12 000 per year)	All
Policy 2: Modified LIFT Act (s_2)	$500 monthly tax credit to adults earning less than $100 000 per year	$500 per month ($6 000 per year)	Individuals with incomes <$100 000 per year
Policy 3: Poverty alleviation (s_3)	All adults are lifted out of poverty, which is defined as having incomes below 100% of the FPL	Individuals with incomes below 100% of the FPL are guaranteed at least 100% of FPL	Individuals with incomes <100% of FPL or <$12 760 per year
Policy 4: NIT (s_4)	Conditional basic income guarantee where adults are guaranteed an income at least 133% of the FPL, and each additional earned income is matched by the government at a rate of 50%	Variable depending on current income; up to 133% in transfers per year	Individuals with incomes <266% of the FPL or <$33 942 per year

FPL, federal poverty level; LIFT, Livable Incomes for Families Today; NIT, negative income tax; UBI, universal basic income.

Policy descriptions

Policy 1: UBI

Policy 1 is universal basic income (UBI), a type of unconditional basic income guarantee, where each adult is given a monthly $1 000 transfer from the government regardless of current income levels.[12] Policy 1 is similar to the "Freedom Dividend" popularized by then-candidate Andrew Yang during the Democratic presidential primaries and to other similar UBI initiatives that have been piloted in selected US cities.[13–16] UBI is likely the most expensive of all the income support and redistributive policies that have been proposed, with estimates ranging from $2.49-3.03 trillion per year depending on the size of the transfer.[12,17] For context, the current cost of existing government transfers in the form of Social Security benefits, Supplemental Nutrition Assistance Program or "food stamps", tax credits such as the Earned Income Tax Credit (EITC), cash welfare, and housing assistance is about $1 trillion annually[17]; when the costs of Medicare, Medicaid, and veterans' benefits are included, the cost is about $2.4 trillion (in 2014 US$).[18]

The income gain from Policy 1 is $12 000 per year per adult. We assumed that there is at least one adult in each household that would receive UBI; as a result, household incomes increase by $12 000 per year. However, this is likely a conservative estimate since more than one qualifying adult may be living in a household, which means some household incomes may increase by at least $24 000 or more per year.

Policy 2: Modified LIFT Act

In Policy 2, we model a smaller monthly transfer of $500 per adult with household incomes less than $100 000 per year; this policy is akin to the LIFT (Livable Incomes for Families Today) Act proposed by Vice President Kamala Harris when she was a senator in the 116th Congress.[19] As a result of Policy 2, eligible adults receive $6 000 annually in income support; in contrast, Vice President Harris's LIFT Act capped the benefit to $3 000 per individual and $6 000 for married couples filing income taxes jointly.

Policy 3: Poverty alleviation

In Policy 3, we simulate a scenario where all US adults are lifted out of poverty. We used the federal poverty level (FPL) for one individual as the threshold for poverty, which in 2019 was set at $12 760 per year.[20] As a result of Policy 3, individuals with annual household incomes below the FPL across all ages and genders move to the next income group where they experience lower all-cause mortality rates. We assume that each eligible individual receives enough government transfer to move them above the FPL.

© 2022 Avanceña ALV et al. JAMA Health Forum.

We do not specify the combination of policies that will lift adults out of poverty, which may include work access programs, minimum wage policies, and housing support.[21] To reduce poverty, experts believe that social welfare programs and programs that encourage employment will have to be implemented concurrently since employment alone will likely be insufficient to eliminate poverty, as evidenced by the 7 million wage-earning adults who live in poverty (so-called "working poor"); additionally, some adults such as the disabled and elderly may not be able to engage in full-time work.[22–24]

© 2022 Avanceña ALV et al. JAMA Health Forum.

Policy 4: NIT

In Policy 4, we simulate a negative income tax (NIT), which a type of conditional basic income guarantee.[24] NIT was first proposed by economist Milton Friedman in his 1962 book *Capitalism and Freedom* as a replacement for various welfare programs, particularly in-kind benefits.[25] NIT gets its moniker from standard income taxes which are monies owed to the government based on income; in contrast, NIT determines an amount owed by the government to an individual or household.

Under NIT, everyone is guaranteed a certain level of income (e.g., 100% of FPL); then, income above this threshold, which is referred to as "earned income", is multiplied by a marginal tax rate (e.g., 50%) that determines the amount of benefit owed by the government. NIT was proposed by the Nixon (i.e., Family Assistance Plan) and Carter administrations but did not receive enough support to pass Congress.[24,26] It is receiving renewed attention as a means to streamline the patchwork of social welfare programs in the US and to more effectively lift people out of poverty while still encouraging them to work.

Under Policy 4, we assumed a threshold income of 133% of FPL ($16 970.80 in 2019) and a marginal tax rate of 50%. With these features, the benefits of NIT phase out at an income level equal to 266% of the FPL (eTable 16).

eTable 16. Effect of a hypothetical negative income tax policies

Earned income level as % of federal poverty level	NIT provided by government as % of federal poverty level	Total income (earned income + NIT) as % of federal poverty level
0	133	133
50	108	158
100	83	183
125	70.5	195.5
150	58	208
175	45.5	220.5
200	33	233
225	20.5	245.5
250	8	258
266	0	266
275	0	275
300	0	300

NIT, negative income tax.

© 2022 Avanceña ALV et al. JAMA Health Forum.

Household income subgroups

Because the population numbers we used are reported by household income group (eTables 5 and 6), we had to assume that individuals in each household income group are uniformly distributed across discrete household income subgroups (eTable 17). This simplifying assumption allowed us to calculate the change in household incomes following each policy scenario more precisely.

eTable 17. Subgroup household incomes (in 2019 US$)

**Household income group (Number of subgroups)**	**Subgroup household income**
Group 1: ≤11 676 (12)	0*
	1 063.27
	2 126.55
	3 189.82
	4 253.09
	5 316.36
	6 379.64
	7 442.91
	8 506.18
	9 569.46
	10 632.73
	11 696.00
Group 2: 11 677-19 915 (9)	11 697.00
	12 724.25
	13 751.50
	14 778.75
	15 806.00
	16 833.25
	17 860.50
	18 887.75
	19 915.00
Group 3: 19 916-27 708 (8)	19 916.00
	21 029.14
	22 142.29
	23 255.43
	24 368.57
	25 481.71
	26 594.86
	27 708.00
Group 4: 27 709-35 348 (8)	27 709.00
	28 800.29
	29 891.57
	30 982.86
	32 074.14
	33 165.43
	34 256.71
	35 348.00
Group 5: 35 349-43 505 (9)	35 349.00
	36 368.50
	37 388.00
	38 407.50
	39 427.00
	40 446.50
	41 466.00
	42 485.50
	43 505.00
Group 6: 43 506-52 493 (9)	43 506.00
	44 629.38
	45 752.75
	46 876.13
	47 999.50
	49 122.88
	50 246.25
	51 369.63
	52 493.00
Group 7: 52 494-63 466 (11)	52 494.00

	53 591.20
	54 688.40
	55 785.60
	56 882.80
	57 980.00
	59 077.20
	60 174.40
	61 271.60
	62 368.80
	63 466.00
Group 8: 63 467-78 567 (16)	63 467.00
	64 473.67
	65 480.33
	66 487.00
	67 493.67
	68 500.33
	69 507.00
	70 513.67
	71 520.33
	72 527.00
	73 533.67
	74 540.33
	75 547.00
	76 553.67
	77 560.33
	78 567.00
Group 9: 78 568-105 574 (28)	78 568.00
	79 568.22
	80 568.44
	81 568.67
	82 568.89
	83 569.11
	84 569.33
	85 569.56
	86 569.78
	87 570.00
	88 570.22
	89 570.44
	90 570.67
	91 570.89
	92 571.11
	93 571.33
	94 571.56
	95 571.78
	96 572.00
	97 572.22
	98 572.44
	99 572.67
	100 572.89
	101 573.11
	102 573.33
	103 573.56
	104 573.78
	105 574.00
Group 10: ≥105 575 (1)	105 575.00

*We conservatively assumed that 0 is the lowest possible household income in our population, as is reported in the ASEC, though in reality households may have negative incomes, such as when they go into debt or own a business with losses.

The number of subgroups was calculated using the formula (Eq. 4)

$$\frac{UL_i - LL_i}{1{,}000} + 1 \text{ (Eq. 4)}$$

© 2022 Avanceña ALV et al. JAMA Health Forum.

where UL_i and LL_i are the upper and lower limits of each household income group (denoted by the index i), respectively, and rounding down the result to the nearest one. For example, in the second household income group (LL_2=$11 677, UL_2=$19 915), the number of subgroups is 9 ((19 915-11 677)/1 000 + 1 = 9.238 ≈ 9). The number of subgroups represents the maximum number of equal increments between the lowest and highest household incomes in each band that are at least $1 000. These increments were the basis of the subgroup household incomes shown in eTable 15, which were the household incomes used to estimate the impact of the modeled policies.

© 2022 Avanceña ALV et al. JAMA Health Forum.

The distribution of the population by household income under Policies 1-4 and the status quo is shown in the eFigure, and the specific changes in household income groups following the implementation of Policies 1-4 are detailed in eTable 18. As both the eFigure and eTable 18 illustrate, Policies 1 and 2 lead to the most shifts in household income and income groups, while Policies 3 and 4 lead to minimal changes.

eFigure. Distribution of the US population by household income under different scenarios

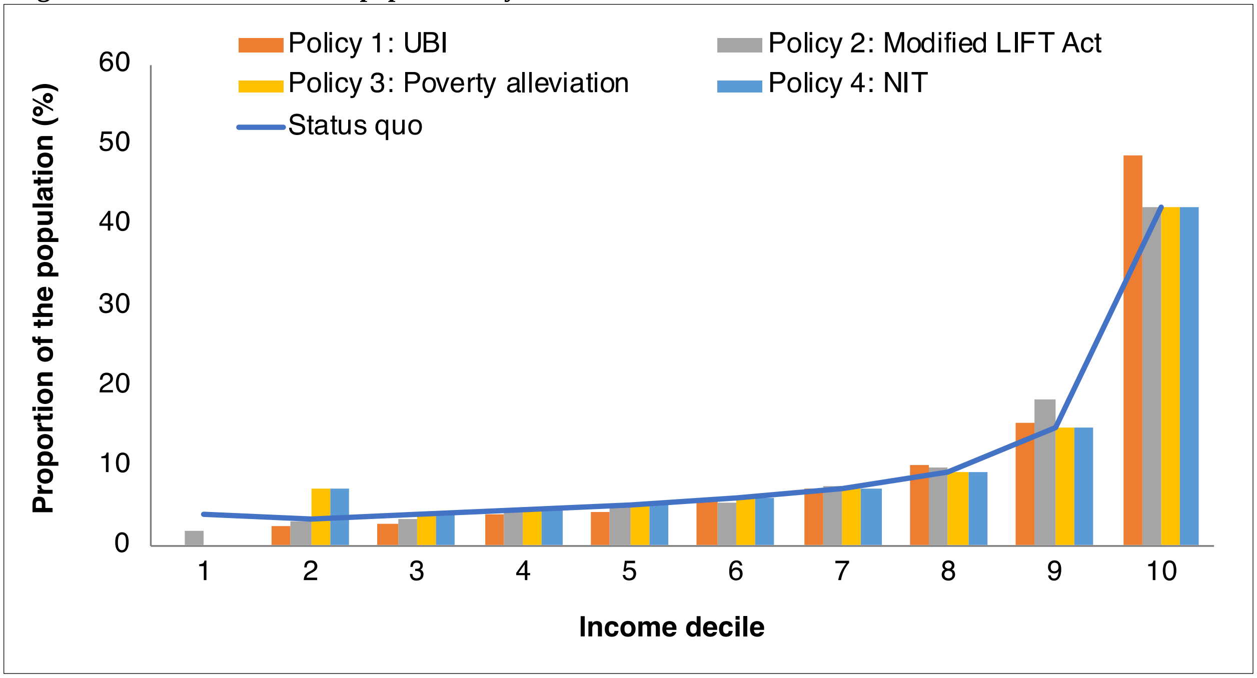

This figure shows the distribution of the US working-age population by annual household income under a status quo scenario (curve) and different hypothetical income support policies (bars).
LIFT, Livable Incomes for Families Today; NIT, negative income tax; UBI, universal basic income.

© 2022 Avanceña ALV et al. JAMA Health Forum.

eTable 18. Transitions between household income groups following implementation of modeled policies

Original household income group	New household income groups after policy (% of population in new group)			
	Policy 1: UBI	*Policy 2: Modified LIFT Act*	*Policy 3: Poverty Alleviation*	*Policy 4: NIT*
1	2 (0.67), 3 (0.33)	1 (0.5), 2 (0.5)	2 (1)	2 (0.5), 3 (0.5)
2	3 (0.44), 4 (0.56)	2 (0.33), 3 (0.67)	2 (1)	3 (1)
3	4 (0.5), 5 (0.5)	3 (0.25), 4 (0.75)	3 (1)	3 (0.25), 4 (0.75)
4	5 (0.5), 6 (0.5)	4 (0.25), 5 (0.75)	4 (1)	4 (1)
5	6 (0.67), 7 (0.33)	5 (0.33), 6 (0.67)	5 (1)	5 (1)
6	7 (0.89), 8 (0.11)	6 (0.33), 7 (0.67)	6 (1)	6 (1)
7	8 (1)	7 (0.45), 8 (0.55)	7 (1)	7 (1)
8	8 (0.25), 9 (0.75)	8 (0.63), 9 (0.38)	8 (1)	8 (1)
9	9 (0.57), 10 (0.43)	9 (1)	9 (1)	9 (1)
10	10 (1)	10 (1)	10 (1)	10 (1)

This table shows the household income groups that individuals move to after the implementation of each modeled policy. For example, after the implementation of UBI, 67% and 33% of those in household income group 1 move to household income groups 2 and 3, respectively. Cells shaded gray are those where no changes in the household income groups are observed between the no-intervention scenario and the modeled policy.
LIFT, Livable Incomes for Families Today; NIT, negative income tax; UBI, universal basic income.

For new entrants to the model, we assumed that their household income distribution is the same as the 18-year-olds that were part of the original simulated cohort.

© 2022 Avanceña ALV et al. JAMA Health Forum.

eMethods, continued

E. Analysis

Our main outcome of interest is deaths averted, which we calculated by taking the difference between the total deaths in the no-intervention scenario and the total deaths from each modeled policy (p_z), as summarized in the following equation (Eq. 5):

$$Deaths\ averted\ by\ p_z = \sum_{i=1}^{10}\sum_{g=1}^{2}\sum_{x=18}^{64}\sum_{y=0}^{64-x} l_{igx+y|s_0} * q_{igx+y} - \sum_{i=1}^{10}\sum_{g=1}^{2}\sum_{x=18}^{64}\sum_{y=0}^{64-x} l_{igx+y|s_a} * q_{igx+y}$$

Sensitivity analysis

Sensitivity analyses allow us to test the influence of our assumptions on the results of our analysis. In this study, we conducted a series of deterministic sensitivity analyses where we varied key assumptions and used different input parameter estimates in the model. First, we varied the distribution of the population by age under the equal and median assumptions discussed previously. Second, we varied the time lag or delay between when a policy is implemented and when individuals experience the mortality rates of their new household income group. Studies on the health effects of income support policies have documented benefits after three years[27–29]; additionally, research on the relationship between income inequality and mortality suggests that the timing of the effect on mortality is not immediate and may vary by population and setting.[30] In the base-case analysis, we assumed that individuals experience the benefit associated with higher household incomes after three years, and in sensitivity analyses, we built in 5-, 10-, and 15-year lags between changes in household income and mortality benefit. With a lag, populations transition to their new household income group but experience the same risk of death as their previous or original household income group until after the lag has lapsed. Third, we used two additional estimates of mortality rates by household income, gender, and age (the high-effect and low-effect scenarios) to generate results for each policy. Fourth, we changed the time horizon to 5, 10, 30, and 40 years, which represent different time horizons that policymakers may use when evaluating income support policies. All-in-all, varying our assumptions in all possible combinations allowed us to generate 180 estimates of deaths averted for each modeled policy and understand the most influential parameters on our results.

F. Limitations

There are several limitations to our study that are worth noting and elaborating. First, we used cross-sectional estimates of the nonlinear association between income gains and mortality, and we assumed that individuals who receive additional income experience reductions in their mortality risk after a lag. While several quasi-experimental and longitudinal cohort studies have shown that higher incomes or increases in income reduce mortality in the US[8,9,27,33,34] and elsewhere[35], there is still disagreement about the magnitude of the effect. Additionally, because the NLMS and PSID focused on adult mortality, we excluded children in our study. Future analyses should also look at the benefit of increased household income on mortality among children, especially since income-based policies like the EITC and raising the minimum wage have both been associated with reductions in infant mortality.[36,37]

Second, we used non-equivalized national household income estimates from ASEC which does not provide additional information on household composition, or the proportion of household income earned from various sources such as wages or government transfers. Thus, in calculating the effect of policies on household income, we had to rely on simplifying assumptions to reasonably estimate the resulting total household income. For example, in modeling the effect of UBI which should benefit every adult aged 18 years and older, we assumed that there was only one adult in each household, leading to a $12 000 annual increase in income. In reality, there may be two or more adults in a household; additionally, household composition may be dynamic over time, which we were not able to model in this study. Similarly, in modeling NIT, we assumed that household income was equal to earned income on which government benefits would be based. Future studies can use income tax return data which provides a more detailed picture of personal and household income, as previous studies have done.[31]

Third, we only modeled household income increases that result from income support policies, and we did not model the effect of potential mechanisms such as progressive taxation that may be used to fund these redistributive policies. For example, UBI may require raising taxes on high-income individuals to implement the policy, and this may shift their household income. However, the policies we modeled may also be funded through other means, such as reallocating existing government funding, reforming existing social safety net programs, or government

© 2022 Avanceña ALV et al. JAMA Health Forum.

borrowing. We also did not model these policies' effect on jobs, prices, and other economic domains, and a "general equilibrium" analysis that considers the effect of income support policies on the whole system may elucidate these issues.

Fourth, we assumed that individuals' household incomes are constant over time, and that any secular changes (i.e., changes not due to the policy) in income will be captured by the income range or bands that individuals are assigned to. This is a major limitation of the study, though it may have not have had a significant effect on our results because of (1) the wide household income bands we used which captures small changes in income over time and (2) research suggesting that intragenerational economic mobility in the US has remained stable/stagnant since the 1980s.[32] Additionally, while there is upward economic mobility, there is also downward economic mobility that may cancel out some of the benefits of the former; for example, between 1994-2004, 22.8% of people in the bottom (poorest) income quintile were able to move up to the second income quintile in 10 years, 21.5% of people who are in the second income quintile moved down to the bottom income quintile over the same time period.[32]

Fifth, we did not model the effect of income inequality on mortality. There is evidence that the magnitude of income inequality—often measured using the Gini coefficient—is positively associated with mortality and other poor health outcomes[30,38–41], and previously published model-based studies exploited this relationship to estimate the health benefits of various income support and redistributive policies.[31,42] However, the literature on income inequality and mortality is mixed, so we opted to exclude it in this analysis. Future studies can explore the effect of both income and income inequality on population health outcomes including mortality.

Finally, we focused exclusively on household income in this study and did not include the effects of other measures of socioeconomic position, such as education, employment, or wealth, which also have documented effects on health outcomes.[42,43] Future studies should look at the independent effects of these social determinants and their intersections on mortality.

© 2022 Avanceña ALV et al. JAMA Health Forum.

eResults

eTable 19. Deaths averted from Policy 1 (Universal basic income) under various assumptions (in thousands)

Time horizon	PSID mortality rates		NLMS mortality rates	
	Equal age assumption	*Median age assumption*	*Equal age assumption*	*Median age assumption*
**3-year lag**				
5-year	302 (221-387)	405 (298-515)	113 (96-124)	152 (129-167)
10-year	954 (707-1208)	912 (674-1157)	371 (315-407)	353 (300-388)
20-year (base-case)	2086 (1564-2602)	2043 (1531-2551)	846 (721-927)	828 (706-907)
30-year	3226 (2443-3982)	3175 (2403-3922)	1338 (1144-1466)	1316 (1125-1441)
40-year	4384 (3346-5365)	4338 (3310-5310)	1841 (1575-2015)	1822 (1558-1994)
**5-year lag**				
10-year	653 (485-821)	507 (376-642)	258 (219-283)	202 (172-221)
20-year (base-case)	1784 (1343-2215)	1639 (1233-2036)	733 (625-803)	676 (578-741)
30-year	2924 (2222-3595)	2770 (2105-3406)	1225 (1048-1341)	1164 (996-1275)
40-year	4082 (3125-4979)	3933 (3012-4794)	1728 (1479-1890)	1670 (1430-1827)
**10-year lag**				
20-year (base-case)	1131 (858-1394)	1131 (857-1394)	475 (406-520)	474 (406-519)
30-year	2271 (1737-2774)	2263 (1729-2765)	968 (828-1059)	963 (824-1053)
40-year	3429 (2639-4157)	3425 (2636-4153)	1470 (1259-1608)	1468 (1258-1606)
**15-year lag**				
20-year (base-case)	561 (427-687)	489 (371-603)	238 (204-261)	204 (174-223)
30-year	1701 (1306-2067)	1621 (1243-1974)	731 (626-799)	692 (593-757)
40-year	2858 (2209-3450)	2784 (2150-3362)	1233 (1057-1348)	1198 (1027-1310)

© 2022 Avanceña ALV et al. JAMA Health Forum.

eTable 20. Deaths averted from Policy 2 (Modified LIFT Act) under various assumptions (in thousands)

Time horizon	PSID mortality rates		NLMS mortality rates	
	Equal age assumption	*Median age assumption*	*Equal age assumption*	*Median age assumption*
**3-year lag**				
5-year	150 (110-192)	200 (147-255)	49 (41-53)	65 (55-72)
10-year	475 (351-602)	454 (335-577)	158 (133-173)	150 (127-165)
20-year (base-case)	1042 (781-1301)	1022 (765-1276)	357 (303-392)	350 (296-383)
30-year	1615 (1223-1994)	1590 (1203-1965)	565 (480-619)	555 (471-608)
40-year	2194 (1674-2687)	2172 (1657-2659)	779 (662-852)	771 (655-843)
**5-year lag**				
10-year	325 (242-409)	254 (188-321)	109 (92-120)	85 (72-93)
20-year (base-case)	892 (672-1108)	821 (618-1020)	309 (262-338)	284 (241-311)
30-year	1465 (1113-1802)	1390 (1056-1709)	516 (439-565)	490 (416-536)
40-year	2045 (1564-2495)	1971 (1509-2404)	731 (621-799)	706 (600-771)
**10-year lag**				
20-year (base-case)	567 (430-699)	567 (430-699)	200 (169-218)	199 (169-218)
30-year	1140 (871-1393)	1136 (868-1388)	407 (346-445)	405 (344-443)
40-year	1719 (1323-2085)	1718 (1321-2083)	621 (529-679)	621 (528-678)
**15-year lag**				
20-year (base-case)	281 (215-345)	246 (186-303)	100 (85-109)	86 (73-94)
30-year	854 (656-1038)	815 (624-992)	308 (262-336)	292 (248-319)
40-year	1434 (1107-1731)	1396 (1078-1687)	522 (445-570)	507 (432-554)

eTable 21. Deaths averted from Policy 3 (Poverty alleviation) under various assumptions (in thousands)

Time horizon	PSID mortality rates		NLMS mortality rates	
	Equal age assumption	*Median age assumption*	*Equal age assumption*	*Median age assumption*
**3-year lag**				
5-year	102 (67-145)	136 (89-190)	36 (28-40)	49 (39-55)
10-year	308 (204-429)	297 (196-415)	112 (90-126)	106 (85-120)
20-year (base-case)	632 (428-861)	621 (420-848)	242 (195-272)	236 (190-266)
30-year	938 (647-1254)	925 (637-1238)	375 (302-421)	368 (297-413)
40-year	1241 (870-1629)	1228 (860-1613)	517 (418-579)	511 (413-572)
**5-year lag**				
10-year	206 (138-284)	161 (106-225)	77 (61-86)	58 (46-65)
20-year (base-case)	530 (362-716)	486 (330-657)	207 (166-232)	188 (151-211)
30-year	836 (580-1109)	790 (547-1048)	339 (274-380)	319 (258-358)
40-year	1139 (803-1484)	1092 (771-1423)	481 (390-539)	462 (374-517)
**10-year lag**				
20-year (base-case)	324 (224-431)	325 (224-432)	130 (105-146)	130 (105-146)
30-year	630 (442-824)	629 (441-823)	263 (213-294)	262 (211-293)
40-year	933 (665-1199)	931 (664-1197)	405 (328-453)	404 (328-452)
**15-year lag**				
20-year (base-case)	157 (109-206)	141 (97-189)	64 (52-72)	55 (45-62)
30-year	463 (328-599)	445 (313-579)	197 (160-220)	187 (151-209)
40-year	765 (551-974)	748 (537-954)	339 (275-379)	330 (268-368)

© 2022 Avanceña ALV et al. JAMA Health Forum.

eTable 22. Deaths averted from Policy 4 (Negative income tax) under various assumptions (in thousands)

Time horizon	PSID mortality rates		NLMS mortality rates	
	Equal age assumption	*Median age assumption*	*Equal age assumption*	*Median age assumption*
**3-year lag**				
5-year	206 (141-280)	277 (191-373)	57 (46-64)	78 (64-87)
10-year	635 (439-852)	608 (419-819)	180 (147-201)	171 (139-190)
20-year (base-case)	1335 (937-1765)	1308 (917-1732)	392 (320-435)	382 (312-425)
30-year	2013 (1430-2629)	1981 (1405-2590)	607 (497-674)	596 (488-661)
40-year	2696 (1936-3482)	2666 (1914-3445)	837 (687-927)	827 (679-916)
**5-year lag**				
10-year	429 (298-572)	331 (228-445)	123 (101-137)	93 (76-103)
20-year (base-case)	1129 (796-1485)	1031 (726-1359)	335 (274-372)	304 (249-338)
30-year	1806 (1289-2348)	1704 (1214-2217)	550 (451-610)	518 (424-574)
40-year	2490 (1795-3202)	2389 (1723-3072)	780 (641-863)	748 (615-829)
**10-year lag**				
20-year (base-case)	700 (498-913)	700 (498-913)	211 (173-235)	211 (173-234)
30-year	1378 (990-1776)	1373 (986-1771)	427 (350-473)	425 (349-471)
40-year	2061 (1497-2630)	2059 (1495-2626)	656 (540-726)	656 (539-725)
**15-year lag**				
20-year (base-case)	342 (245-442)	301 (213-394)	105 (86-116)	90 (73-100)
30-year	1019 (737-1306)	974 (702-1252)	320 (263-354)	303 (249-336)
40-year	1703 (1243-2159)	1659 (1210-2107)	550 (453-608)	534 (440-591)

© 2022 Avanceña ALV et al. JAMA Health Forum.

eReferences

1 U.S. Census Bureau. Current Population Survey design and methodology. Washington, D.C.: U.S. Census Bureau, 2019.

2 U.S. Census Bureau. Annual Social and Economic Supplement (ASEC) of the CPS. The United States Census Bureau. 2020; published online Dec 7. https://www.census.gov/programs-surveys/saipe/guidance/model-input-data/cpsasec.html (accessed Jan 6, 2021).

3 U.S. Census Bureau. Current Population Survey 2020 Annual Social and Economic (ASEC) Supplement. 2020. https://www2.census.gov/programs-surveys/cps/techdocs/cpsmar20.pdf (accessed Jan 5, 2021).

4 U.S. Census Bureau. Household Income: HINC-03. 2020. https://www.census.gov/data/tables/time-series/demo/income-poverty/cps-hinc/hinc-03.html (accessed Jan 6, 2021).

5 Brodish PH, Hakes JK. Quantifying the individual-level association between income and mortality risk in the United States using the National Longitudinal Mortality Study. *Social Science & Medicine* 2016; **170**: 180–7.

6 Dowd JB, Albright J, Raghunathan TE, Schoeni RF, LeClere F, Kaplan GA. Deeper and wider: income and mortality in the USA over three decades. *International Journal of Epidemiology* 2011; **40**: 183–8.

7 Consumer Price Index (CPI) Databases : U.S. Bureau of Labor Statistics. https://www.bls.gov/cpi/data.htm (accessed May 25, 2021).

8 Products - Life Tables - Homepage. 2021; published online April 14. https://www.cdc.gov/nchs/products/life_tables.htm (accessed May 25, 2021).

9 Hoynes H, Rothstein J. Universal basic income in the United States and advanced countries. *Annu Rev Econ* 2019; **11**: 929–58.

10 Yang A. The Freedom Dividend. Yang2020 - Andrew Yang for President. 2020. https://www.yang2020.com/policies/the-freedom-dividend/ (accessed Oct 7, 2020).

11 The Associated Press. Mayors vow to launch guaranteed income programs across US. CBS Sacramento. 2020; published online Sept 16. https://sacramento.cbslocal.com/2020/09/16/ubi-guaranteed-income-programs-stockton/ (accessed Oct 9, 2020).

12 Holder S. 2021 will be the year of guaranteed income experiments. Bloomberg.com. 2021; published online Jan 4. https://www.bloomberg.com/news/articles/2021-01-04/guaranteed-income-gains-popularity-after-covid-19 (accessed Jan 6, 2021).

13 Lowrey A. Stockton's basic-income experiment pays off. The Atlantic. https://www.theatlantic.com/ideas/archive/2021/03/stocktons-basic-income-experiment-pays-off/618174/ (accessed March 13, 2021).

14 Kearney MS, Mogstad M. Universal basic income (UBI) as a policy response to current challenges. 2019; published online Aug 23. https://www.brookings.edu/wp-content/uploads/2019/08/UBI-ESG-Memo-082319.pdf (accessed Oct 9, 2020).

15 Ensor W, Frailey A, Jensen M, Xu A. A budget-neutral universal basic income. Washington, D.C.: American Enterprise Institute, 2017.

16 Maag E. Senator Kamala Harris proposed a bold tax credit to help low- and middle-income workers. Tax Policy Center. 2020; published online Aug 13. https://www.taxpolicycenter.org/taxvox/senator-kamala-harris-proposed-bold-tax-credit-help-low-and-middle-income-workers (accessed April 6, 2021).

© 2022 Avanceña ALV et al. JAMA Health Forum.

17 U.S. Centers for Medicare & Medicaid. Federal Poverty Level (FPL). https://www.healthcare.gov/glossary/federal-poverty-level-fpl/ (accessed Jan 6, 2021).

18 Ellwood DT, Patel NG. Restoring the American dream. 2018; published online Jan. https://www.mobilitypartnership.org/restoring-american-dream (accessed Jan 5, 2021).

19 Nightingale DS, Loprest PJ. Work alone is often not enough to lift people out of poverty. Urban Institute. 2018; published online April 13. https://www.urban.org/urban-wire/work-alone-often-not-enough-lift-people-out-poverty (accessed Jan 6, 2021).

20 U.S. Bureau of Labor Statistics. A profile of the working poor, 2018. 2020; published online July. https://www.bls.gov/opub/reports/working-poor/2018/home.htm (accessed Jan 6, 2021).

21 Wiederspan J, Rhodes E, Shaefer HL. Expanding the discourse on antipoverty policy: reconsidering a negative income tax. *Journal of Poverty* 2015; **19**: 218–38.

22 Friedman M. Capitalism and freedom. Chicago: The University of Chicago Press, 1962 https://www.google.com/books/edition/Capitalism_and_Freedom/zHSv4OyuY1EC?hl=en&gbpv=0.

23 Levine RA, Watts H, Hollister R, Williams W, O'Connor A, Widerquist K. A retrospective on the negative income tax experiments: looking back at the most innovate field studies in social policy. In: Widerquist K, Lewis MA, Pressman S, eds. The Ethics and Economics of the Basic Income Guarantee, 1st edn. London: Routledge, 2005: 95–106.

24 Zheng H. Do people die from income inequality of a decade ago? *Social Science & Medicine* 2012; **75**: 36–45.

25 Kim D. Projected impacts of federal tax policy proposals on mortality burden in the United States: a microsimulation analysis. *Preventive Medicine* 2018; **111**: 272–9.

26 Acs G, Zimmerman S. U.S. intragenerational economic mobility from 1984 to 2004. 1984. https://www.urban.org/sites/default/files/publication/31316/1001226-u-s-intragenerational-economic-mobility-from-to-.pdf.

27 Muennig P, Vail D, Hakes JK. Can antipoverty programmes save lives? Quasi-experimental evidence from the Earned Income Tax Credit in the USA. *BMJ Open* 2020; **10**: e037051.

28 Cristia JP. The empirical relationship between lifetime earnings and mortality. 2011. https://www.cbo.gov/sites/default/files/110th-congress-2007-2008/workingpaper/2007-11_0.pdf (accessed Oct 14, 2019).

29 Lantz PM, Golberstein E, House JS, Morenoff J. Socioeconomic and behavioral risk factors for mortality in a national 19-year prospective study of U.S. adults. *Social Science & Medicine* 2010; **70**: 1558–66.

30 Kinge JM, Modalsli JH, Øverland S, *et al.* Association of household income with life expectancy and cause-specific mortality in Norway, 2005-2015. *JAMA* 2019; **321**: 1916.

31 Strully KW, Rehkopf DH, Xuan Z. Effects of prenatal poverty on infant health: state Earned Income Tax Credits and birth weight. *Am Sociol Rev* 2010; **75**: 534–62.

32 Komro KA, Livingston MD, Markowitz S, Wagenaar AC. The effect of an increased minimum wage on infant mortality and birth weight. *Am J Public Health* 2016; **106**: 1514–6.

33 Bor J, Cohen GH, Galea S. Population health in an era of rising income inequality: USA, 1980–2015. *The Lancet* 2017; **389**: 1475–90.

© 2022 Avanceña ALV et al. JAMA Health Forum.

34 Pickett KE, Wilkinson RG. Income inequality and health: a causal review. *Social Science & Medicine* 2015; **128**: 316–26.

35 Blakely TA. What is the lag time between income inequality and health status? *Journal of Epidemiology & Community Health* 2000; **54**: 318–9.

36 Kim D. The associations between US state and local social spending, income inequality, and individual all-cause and cause-specific mortality: The National Longitudinal Mortality Study. *Preventive Medicine* 2016; **84**: 62–8.

37 Galea S, Tracy M, Hoggatt KJ, DiMaggio C, Karpati A. Estimated deaths attributable to social factors in the United States. *Am J Public Health* 2011; **101**: 1456–65.

38 Backlund E, Sorlie PD, Johnson NJ. A comparison of the relationships of education and income with mortality: the national longitudinal mortality study. *Social Science* 1999; : 12.

© 2022 Avanceña ALV et al. JAMA Health Forum.
